# How well can commonly used anxiety scales detect treatment outcomes in the context of autism?

**DOI:** 10.1177/13623613251349929

**Published:** 2025-07-05

**Authors:** Huilin Chen, Jeffrey J Wood, Connor M Kerns, Eric A Storch, Philip C Kendall, Gaia Scerif, Cathy Creswell

**Affiliations:** 1 University of Oxford, UK; 2 University of California, Los Angeles, USA; 3 The University of British Columbia, Canada; 4 Baylor College of Medicine, USA; 5 Temple University, USA

**Keywords:** anxiety, autism, measurements, treatment outcomes

## Abstract

Anxiety is one of the most prevalent mental health challenges in autistic children, yet there is limited evidence on effective tools to measure treatment outcomes. Previous research with non-autistic children has found that the Child Anxiety Impact Scale, Parent Version achieved good diagnostic accuracy when measuring treatment outcomes and performed better than a commonly used symptom measure. However, this has not been evaluated for autistic children. The present study examined the psychometric properties of the Child Anxiety Impact Scale, Parent Version in autistic children and compared its utility against other anxiety symptom measures, to detect treatment outcomes as assessed by a gold-standard diagnostic interview, the Anxiety and Related Disorders Interview Schedule, Child Version, Parent Interview with the Autism Spectrum Addendum. Data were used from 212 children (aged 7–13 years) who participated in a randomised controlled trial. Receiver-operating characteristic curve analyses were conducted, and subsequent subgroup analyses were conducted using DeLong tests. Results demonstrated that the Child Anxiety Impact Scale, Parent Version had strong psychometric properties, with total scores significantly outperforming other measures in predicting post-treatment recovery from anxiety diagnoses. These findings have implications for future choices of treatment outcome measures in research and clinical practice.

**Lay abstract:**

**Study on the utility of anxiety scales to detect anxiety diagnostic treatment outcomes in autistic children**

**Why was the study done?** The importance of having valid and reliable anxiety measures for autistic children has been highlighted as a research priority by professionals and people with lived experience. Yet, while anxiety has been frequently assessed in autistic children, we do not currently know much about how well commonly used anxiety measures work, especially parent reports, in this context. This has significant implications for care planning and resource allocation for autistic children who experience significant anxiety problems.

**What did the researchers do?** The research team studied data collected in a previously published multi-centred randomised controlled trial (RCT) testing an adapted cognitive behavioural therapy for anxiety (Wood et al., 2020) to better understand how different anxiety measures did, compared to gold-standard anxiety diagnostic assessments, in detecting treatment outcomes. They focused in particular on the Child Anxiety Impact Scale, Parent Version (CAIS-P).

**What did the researchers find?** This study found that the CAIS-P did better than conventional anxiety symptom measures in detecting treatment outcomes for anxiety problems in autistic children.

**What do the findings mean?** This study adds to the current evidence base to inform choices of measurement of anxiety problems in the context of autism.

## Introduction

Anxiety problems are among the most common mental health problems reported (Lai et al., 2019) and treated (Zablotsky et al., 2015) in the context of autism, especially for children and young people. One meta-analysis found a pooled prevalence of 39.7% of diagnosable anxiety problems in autistic children and young people (Van Steensel et al., 2011). Similarly, a large-scale national cohort study (*N* = 2077; Zablotsky et al., 2015) identified that 41.1% of autistic children and young people (without intellectual disability) experienced clinical levels of anxiety. Prevalence rates may be even higher when autism-related expressions of anxiety are also considered. This could include fears related to change, sensory differences and social uncertainty (Den Houting et al., 2019; Kerns et al., 2014, 2021). Unsurprisingly, given this high prevalence, anxiety problems have been prioritised as a research target by people with lived experience of autism and professionals working with autistic people, including the need for better measurement of mental health needs including anxiety problems (Benevides et al., 2020).

Diagnostic interviews are the gold-standard method for assessing treatment outcomes for anxiety problems; one commonly used example is the Anxiety Disorders Interview Schedule for children (ADIS-C). Recently, the ADIS with autism spectrum addendum (ADIS/ASA, Kerns et al., 2017) has been developed as an autism-tailored version for the ADIS-C. The ADIS/ASA provides guidelines for differentiation of overlapping autism and anxiety-related behaviours (e.g. social avoidance, rigidity) and examines presentations of anxiety that can arise within the context of autism that are not assessed within the ADIS-C. The ADIS/ASA has been validated in autistic children, with strong inter-rater and test–retest reliability, and has been shown to successfully address diagnostic overshadowing (Kerns et al., 2017). However, due to the time and level of training required, structured diagnostic interviews are rarely used to assess treatment outcomes in practice outside randomised controlled trials (RCTs) (Creswell et al., 2020). Instead, clinicians have often relied on self- or parent-report questionnaire measures as a more feasible method to assess treatment outcomes. While there is evidence that commonly used measures are capable of predicting remission or response to anxiety treatment in non-autistic children (Caporino et al., 2017; Evans et al., 2017; Palitz et al., 2018), it is unclear to what extent questionnaire measures accurately reflect diagnostic treatment outcomes for autistic children.

Notably, in studies with non-autistic children, questionnaires assessing interference associated with anxiety have fared better than symptom measures when it comes to predicting diagnostic outcomes, particularly when parent report is used. For example, the parent-reported Child Anxiety Impact Scale (CAIS-P, Langley et al., 2014) had better accuracy (area under the curve, AUC = 0.81) in detecting recovery from all anxiety diagnoses among 7- to 12-year-old children than the Spence Children’s Anxiety Scale, parent report (SCAS-P, AUC = 0.75) and was the only measure that achieved an AUC above 0.70 for detecting recovery from primary anxiety diagnoses (Evans et al., 2017). However, it is unclear whether findings will be similar when measuring responses to treatment of anxiety problems in autistic children, particularly given the fact that most commonly used measures were developed and validated with non-autistic populations. Indeed, there is evidence that, when applied with autistic children, a number of commonly used measures of anxiety have reduced accuracy in detecting anxiety disorders, due to differences in respondents’ interpretation of items, differential relevance across different constructs being evaluated and differences in factorial structure (Creswell et al., 2020; Glod et al., 2017; Kerns et al., 2015, 2021; White et al., 2015). Furthermore, presentations of anxiety that may be related to autism (e.g. relating to social communication differences, focused interests, sensory processing differences and anxiety around routines) may not necessarily be captured by conventional tools (Kerns et al., 2015, 2021; Kerns & Kendall, 2012). There are also concerns about reduced interoceptive accuracy and higher subjective confidence for interoceptive judgement in autistic children (Palser et al., 2018), which may affect responses to commonly used measures including by parents (DuBois et al., 2016). As such, it is essential that the psychometric properties of commonly used measures to assess treatment progress are evaluated among autistic children, including establishing the extent to which they can predict treatment outcomes as assessed by a gold-standard diagnostic interview and how this may vary in the context of different characteristics of the child and their experience of anxiety and autism. Moreover, given that the focus of fears may be broader for autistic youth, questionnaires focused on the impairment arising from anxiety symptoms (regardless of their foci) rather than the specific quality of anxiety symptoms may have advantages.

Furthermore, the appropriateness of a given anxiety measure for autistic children and young people could vary across clinical and sociodemographic profiles. In terms of age appropriateness and verbal abilities, some anxiety measures such as the Revised Child Anxiety and Depression Scale (RCADS) might be more suitable for older, or more verbal autistic children (Lecavalier et al., 2014). However, little is known about the performance of anxiety measures in under-represented subgroups of autistic children and young people, especially those with more complex comorbid anxiety profiles, higher levels of autistic traits, girls and those of minority racial (global majority) backgrounds, given the inherent sampling imbalance in autism research with most participants being high functioning, male and White individuals.

Receiver-operating characteristic (ROC) curves are useful in examining the diagnostic accuracy of scale measures in predicting treatment outcomes. An ROC simulates the process of clinical diagnostic practice where the evaluation is focused on assessing the extent to which cases with a certain problem are categorised (or diagnosed) accurately, and those without the problem are not categorised (or diagnosed) accidentally (Youngstrom, 2014). This is important when it comes to measuring treatment progress in the context of autism, as assessment outcomes can have important implications for the support and services that an autistic child and young person will get.

## Aims and objectives

Given the importance of having parent-report measures of child anxiety that are valid and reliable for measuring treatment outcomes for autistic children, this study aimed to examine the performance of the CAIS-P, and other anxiety symptom measures, with a specific focus on their ability to detect diagnostic treatment outcomes, using data from an established randomised controlled trial, the Treatment of Anxiety in Autism Spectrum Disorder (TAASD) trial (Wood et al., 2020).

This study has two primary research questions: (1) Does the parent-reported Child Anxiety Impact Scale-Parent (CAIS-P) have satisfactory psychometric properties, in particular, internal consistency, convergent, factor and construct validities for autistic children at pre- and post-treatment? And (2) Does the parent-reported CAIS-P have satisfactory diagnostic accuracy for detecting recovery from anxiety disorders, and how does the diagnostic accuracy of CAIS-P compare to that of other commonly used scales?

In addition, the study will explore the following secondary research question: Does the diagnostic accuracy of different measures differ according to different individual characteristics, including children’s autism traits, baseline comorbidities of anxiety disorders, children’s verbal ability and sociodemographic variables including children’s age, sex and race?

## Methods

### Pre-registration of protocol

A protocol was pre-registered on the Open Science Framework.^1^

### Participants

Two hundred and twelve participants from Wood et al. (2020) aged 7 to 13 years old (*M* = 9.99 years, *SD* = 1.77 years) were included in this study (Table 1). This is the number of children that were screened at baseline for participation. Only the 167 that were eligible and randomised (and so were offered treatment and outcome assessments) were included in the ROC analyses (see Supplemental Appendix 1 for a descriptive summary on sample diagnostic characteristics).

**Table 1.** Sample sociodemographic characteristics.

	Sample distribution	
Sociodemographic variables	*N*	Percentage
Age range	212	
7–9 years old	95	44.81%
10–13 years old	117	55.19%
Sex	212	
Female	45	21.23%
Male	167	78.77%
Race	209	
Black or African American	12	5.74%
Asian	15	7.18%
White	162	77.51%
Native Hawaiian or Other Pacific Islander	1	0.48%
American Indian or Alaskan Native	3	1.44%
Others	16	7.66%
	*N*	Mean (standard deviation) Percentage
Full-scale IQ	204	100.97 (15.94)
SRS-2	200	159.49 (20.77)

Autism spectrum diagnoses were confirmed by trained and research-certified assessors during the recruitment process of the trial using the Childhood Autism Rating Scale Second Edition–High Functioning Version (Schopler et al., 2010) and the Autism Diagnostic Observation Schedule–2 (ADOS-2, Lord et al., 2000). In addition to an autism spectrum diagnosis, the eligibility criteria of the original trial (Wood et al., 2020) include (1) an IQ of or above 70 (± SEM) and (2) significant anxiety problems as defined by a Paediatric Anxiety Rating Scale (PARS) total score of or above 14.

Of those who reported information on their race (*N* = 209), the majority were racially White (*N* = 162, 76.1%), 15 identified as Asian (7.0%), 12 identified as Black or African American (5.6%) and 1 participant identified as Native Hawaiian or Other Pacific Islander (0.5%). 63.4% of participants came from families with medium to high household incomes of $70,000 or above. Details on full sample characteristics and intervention procedures have previously been published (Wood et al., 2020).

### Measures

#### Anxiety and Related Disorders Interview Schedule, Child Version, Parent Interview with the Autism Spectrum Addendum

Anxiety diagnoses were made using the Anxiety and Related Disorders Interview Schedule, Child Version, Parent Interview with the Autism Spectrum Addendum (ADIS/ASA) (Kerns et al., 2017, 2014). As previously discussed, the ADIS/ASA was developed and validated specifically for autistic children and satisfactory psychometric properties have been established, with good inter-rater reliability (intraclass correlation (ICC) = 0.85–0.98, κ = 0.67–0.91, Kerns et al., 2017). The ADIS/ASA Clinician Severity Ratings have previously shown good convergent validity with various anxiety assessments, including PARS and Child Behaviour Checklist (CBCL) *DSM* anxiety problems subscale, while maintaining divergent validity with CBCL attention and aggression subscales (Kerns et al., 2017). Cross-site training on the administration of the ADIS/ASA was directed by a senior researcher experienced in assessing and treating anxiety problems in autistic children and young people (C.M.K.). Raters were blind to treatment allocation and were trained to research-reliable level (i.e. scored within 1 point on the ADIS/ASA severity rating and 100% diagnostic agreement) with C.M.K. or another already reliable clinician. As previously reported, good inter-rater reliability has been found from a subset of the current sample for both the principal diagnoses (κ = 0.82), and the presence and absence of *DSM* (κ = 0.67–0.91) and non-*DSM* anxiety diagnoses (κ = 0.67–0.91, Kerns et al., 2017).

#### Child Anxiety Impact Scale, Parent Report

The CAIS-P was developed to measure anxiety-related functional impairments in the context of school, social and family life (Langley et al., 2014). Parents were asked to rate the degree to which anxiety interfered with 27 activities on a 4-point Likert-type scale (‘0’ not at all, ‘1’ just a little, ‘2’ pretty much, ‘3’ very much) across the three contexts, for example, ‘Getting to School on Time’. It was validated in 488 non-autistic children aged between 7 and 17 years old, with good structural validity, internal consistency and construct validity (Langley et al., 2014). As previously discussed, the CAIS-P was shown to have satisfactory diagnostic accuracy in detecting recovery in non-autistic children with anxiety disorders (Evans et al., 2017).

#### Paediatric Anxiety Rating Scale

The PARS is a clinician-administered parent-report measure that provides an assessment of the number of anxiety symptoms, symptom frequency, distress, physical symptoms, avoidance, home interference and other interference associated with child anxiety (Research Units on Pediatric Psychopharmacology Anxiety Study Group, 2002). The clinician rated the level of concerns across the seven dimensions on a 6-point scale (‘0’ none, ‘1’ minimal to ‘5’ extreme). The PARS has been shown to have promising sensitivity and specificity at an optimal cutoff for assessing treatment outcomes in non-autistic population (Johnco et al., 2015). In the context of autism, the PARS had shown good test–retest and inter-rater reliability, but weaker internal consistency (Cronbach’s alpha = 0.59, Storch et al., 2012). Separate normative data for boys and girls are available for the PARS. Raw scores are used in this study.

#### Child Behaviour Checklist 6–18, Parent Report, Anxious/Depressed Subscale

The CBCL Anxious/Depressed subscale is a 13-item subscale frequently used to examine internalising symptoms in children and young people (Achenbach & Rescorla, 2001). Parents rated the child’s behaviours (e.g., ‘cries a lot’) on a 3-point scale from 0 to 2, with higher scores indicating more severe problems. Satisfactory psychometric properties of the CBCL were reported based on a study of 122 autistic youth, aged between 6 and 18 years (Pandolfi et al., 2012). The anxious/depressed subscale was found to have satisfactory diagnostic accuracy (AUC = 0.724), with good sensitivity (0.914) but relatively low specificity (0.446) at the optimal cutoff for classifying the presence or absence of anxiety disorders (Pandolfi et al., 2012). Separate normative data for boys and girls are available for CBCL. Raw scores are used in this study.

#### Multidimensional Anxiety Scale for Children, Parent Version

The Multidimensional Anxiety Scale for Children, Parent Version (MASC-P) is a measure of anxiety symptoms comprised of 39 items with the following subscales: harm avoidance, physical symptoms, social anxiety and separation anxiety/panic, with satisfactory psychometric properties in non-autistic children (March et al., 1997; Rynn et al., 2006). Items relating to anxiety symptoms (e.g., ‘Feels tense’) were scored on a 4-point scale from 0 (Never true about my child) to 3 (Always true about my child). The MASC-P total score has been shown to have satisfactory diagnostic accuracy on mother report (AUC = 0.70) and moderate diagnostic accuracy on father report (AUC = 0.64, Villabø et al., 2012) in non-autistic children. Notably, the MASC-P has been shown to have a modestly different measurement characteristics in a confirmatory factor model among autistic children compared to that found among non-autistic children (White et al., 2015), despite good psychometric properties have also been reported in youth with learning disability (Thaler et al., 2010). Separate normative data for boys and girls are available for MASC-P. Raw scores were used in this study.

#### Social Responsiveness Scale–2, Parent Version

The extent of autism traits was measured using the Social Responsiveness Scale–2, Parent Version (SRS), which has been found to have good sensitivity and specificity for screening for autistic traits in clinical settings (Bölte et al., 2011; Constantino, 2002). Parents were required to rate 65 items on their child’s behaviours (e.g. ‘Plays appropriately with children his or her age’) over the past 6 months from 1(Not true) to 4 (Almost always true). A higher score represents higher levels of autism traits. The SRS-2 was found to be sensitive (0.92) and specific (0.92) in predicting an autism diagnosis, with satisfactory psychometric properties (Bruni, 2014).

#### Wechsler Intelligence Scale for Children IV, Vocabulary Subscale

The Vocabulary Subscale of the Wechsler Intelligence Scale for Children IV (WISC) was used as an indicator of verbal ability (Wechsler, 2003). It has been frequently used with autistic children and young people with average or above average IQ (Mayes & Calhoun, 2008; Oliveras-Rentas et al., 2012).

### Data analysis and missing data

All analyses were conducted in accordance with our pre-registered protocol (except for the specified changes^2^) using R studio Version 4.3.3. Initial visual inspections, assumption testing and confirmatory factor analysis (CFA) were conducted using SPSS, Version 29.0.1 and AMOS Version 28.

To assess the psychometric properties of the CAIS-P for use with autistic children (research question 1), the internal consistency of the CAIS-P was examined by calculating Cronbach’s alphas pre- and post-treatment and the convergent validity of the CAIS-P with other symptom measures was examined using Spearman’s rho analyses. Correlations between CAIS-P indices and functioning within and outside school settings, as indicated by the CBCL-social competence subscale, were also carried out. These analyses provide insights into whether academic and social performance (as measured by the CBCL-social competence subscale) are associated with the impact of anxiety in school and social contexts (as measured by the CAIS-P). To assess the construct validity of the CAIS-P social and school subscales in relation to autistic children’s social functioning, Spearman’s rho analyses were conducted, given the non-parametric nature of the data (Supplemental Appendix 2). Finally, subscale scores for non-autistic children on the CAIS-P are computed on the basis of an acceptable fit of a three-factor model for the original non-autistic validation sample. Therefore, a CFA based on the original three-factor model in the non-autistic validation sample was conducted, to explore whether similar subscale scores should be used. Available data from all 212 participants were included in this analysis.

To address research question 2, diagnostic accuracy was analysed by calculating the AUC of ROCs for the four anxiety symptom and impact measures (CAIS-P, PARS, CBCL, MASC-P) for classifying recovery from primary or all anxiety diagnoses (including both *DSM* anxiety diagnoses and other impairing anxiety assessed by the ADIS/ASA) post-treatment. We included both of these diagnostic outcomes as they are both commonly used in reporting child treatment outcomes in anxiety treatment trials (Creswell et al., 2021). For our analyses, recovery from primary anxiety diagnoses was defined by a post-treatment Clinician Severity Rating (CSR) < 4 as standard for the primary (or most impairing) anxiety disorders with the highest baseline CSR as assessed by ADIS/ASA. Recovery from all anxiety diagnoses was defined by CSR < 4 as standard for all anxiety diagnoses on ADIS/ASA.

In order to address the extent to which diagnostic accuracy varies across measures and with individual characteristics of the child (the exploratory research question), previously calculated ROCs of each measure were compared using paired DeLong Tests. Variables of interest including children’s autism traits, number of baseline anxiety comorbidities, verbal abilities, sex and age were then used to create subgroups to compute ROCs for each subgroup (see Supplemental Appendix 8 for details). Unpaired DeLong Test was conducted for subgroup comparisons. Subgroups of all variables of interest except for verbal ability, age and race^3^ were created for children with respective scores in top 30%, middle 40% and bottom 30% of the total sample, to capture the variability across the sample, based on data distribution. Only data from the 167 participants who were randomised and received treatment were used for analyses for research question 2 and the secondary research question.

Completion rates for the CAIS-P among the available population were 62.4%. Little’s Missing Completely At Random Test revealed a not missing completely at random structure, $\chi^{2}\left(46\right)=224.24,p<0.001$ (see Supplemental Appendix 3 for detailed examination of missing data pattern). For our main analyses, we performed complete case analyses with pair-wise deletion. However, in light of the missingness, we also repeated all our analyses using missForest imputation (Stekhoven & Bühlmann, 2012), the full results of which can be found in Supplemental Appendix 4. The findings from the two analyses were broadly similar; we therefore refer mainly to our complete case analyses in the Results section and note where inconsistency occurs between the complete case analyses and imputed analyses separately.

## Results^4^

### Pre-registered analyses

#### Research Question 1: psychometric properties and factor structure of the CAIS-P in the context of autism

In the current sample, the CAIS-P was found to have satisfactory internal consistency both pre-treatment (Cronbach’s α = 0.854) and post-treatment (Cronbach’s α = 0.884, see Supplemental Appendix 2 for Cronbach’s α of other scales). Likewise, good convergent validity was found between CAIS-P, CBCL-anxious/depressed, MASC-P and PARS, pre- and post-treatment; all measures were significantly and at least moderately (Schober et al., 2018) inter-correlated pre- and post-treatment (Table 2).

**Table 2.** Convergent validity of measures.

Measures	Pre-treatment		Post-treatment	
	Spearman’s rho	Significance level	Spearman’s rho	Significance level
CAIS-P & CBCL-Anxious/Depressed	0.440	***p*** **<** 0.001^**^	0.498	***p*** **<** 0.001^**^
CAIS-P & MASC-P	0.420	***p*** **<** 0.001^**^	0.408	***p*** **<** 0.001^**^
CAIS-P & PARS	0.344	***p*** **<** 0.001^**^	0.533	***p*** **<** 0.001^**^

CAIS-P: Child Anxiety Impact Scale, Parent Report; CBCL-Anxious/Depressed subscale; MASC-P: Multidimensional Anxiety Scale for Children, Parent Version; PARS: Paediatric Anxiety Rating Scale severity mean score.

** Correlation is significant at confidence level after Bonferroni correction: *p* = 1 − 0.05/6 = 0.992.

Spearman’s rho correlations between the CAIS social impact and CBCL-social competence subscale pre- or post-treatment were weak and nonsignificant, and weak and only trend significance was found between the CAIS school impact and CBCL-social competence subscale, pre-treatment ( ρ = −0.149, Bonferroni-adjusted *p* = 0.032, see Supplemental Appendix 5 for full results) and post-treatment ( ρ = −0.180, Bonferroni-adjusted *p* = 0.037, see Supplemental Appendix 5 for full results) on the complete case dataset. Likewise, weak and trend significant correlations were found between CAIS social impact subscale and CBCL-social competence subscale post-treatment ( ρ = −0.149, Bonferroni-adjusted *p* = 0.030, see Supplemental Appendix 4 for full results), and between CAIS school impact subscale and CBCL-social competence subscale before ( ρ = −0.136, Bonferroni-adjusted *p* = 0.047, see Supplemental Appendix 4 for full results) and after treatment ( ρ = −0.180, Bonferroni-adjusted *p* = 0.009, see Supplemental Appendix 4 for full results) with imputation after Bonferroni correction.

The CFA was conducted to assess whether subscale scores generated on the basis of a non-autistic validation sample were warranted here. CFA revealed poor fit of the current CAIS-P data to the original three-factor structure before treatment, $\chi^{2}\left(345\right)$ = 1129.79, *p* < 0.001, with a low comparative fit index (CFI) of 0.526, and medium to high levels of error as measured by root mean square error of approximation (RMSEA) of 0.108 (see Supplemental Appendix 6 for details of latent factor loadings). A similar pattern was also observed with the post-treatment CAIS-P data, where poor fit to the original three-factor structure was found, $\chi^{2}\left(321\right)$ = 917.25, *p* < 0.001, CFI = 0.566, RMSEA = 0.120. Subsequent analyses were therefore only conducted using the total scores of CAIS-P, without investigating specific subscales.

#### Research Question 2.1: diagnostic accuracy for detecting full recovery

The CAIS-P was the only measure that achieved satisfactory diagnostic accuracy (as defined by an AUC ⩾ 0.7) for predicting recovery from all anxiety diagnoses after treatment in either the complete case analyses (Table 3) or with imputations (Supplemental Appendix 4).

**Table 3.** The diagnostic accuracy of CAIS-P, CBCL-anxious/depressed, MASC-P and PARS-Severity for detecting full recovery post-treatment.

Measures	Diagnostic accuracy (area under the curve)
CAIS-P	0.802
CBCL-Anxious/Depressed	0.533
MASC-P	0.542
PARS	0.496

CAIS-P: Child Anxiety Impact Scale, Parent Report; CBCL-Anxious/Depressed subscale; MASC-P: Multidimensional Anxiety Scale for Children, Parent Version; PARS: Paediatric Anxiety Rating Scale severity mean score.

#### Research Question 2.2: diagnostic accuracy for detecting recovery from primary diagnoses

No measure had satisfactory diagnostic accuracy (as defined by an AUC ⩾ 0.7) for predicting recovery from primary anxiety diagnoses post-treatment in either the complete case analyses (Table 4) or with imputations (Supplemental Appendix 4).

**Table 4.** The diagnostic accuracy of CAIS-P, CBCL-anxious/depressed, MASC-P and PARS-Severity for detecting recovery from primary anxiety diagnoses post-treatment.

Measures	Diagnostic accuracy (area under the curve)
CAIS-P	0.536
CBCL-Anxious/Depressed	0.657
MASC-P	0.603
PARS	0.477

CAIS-P: Child Anxiety Impact Scale, Parent Report; CBCL-Anxious/Depressed subscale; MASC-P: Multidimensional Anxiety Scale for Children, Parent Version; PARS: Paediatric Anxiety Rating Scale severity mean score.

### Exploratory analysis

#### Subgroup comparisons of diagnostic accuracy

To address the exploratory research question, paired DeLong tests were conducted to compare the diagnostic performance of the four measures in terms of predicting primary and full recovery post-treatment. Bonferroni correction was applied to the *p*-values to account for multiple comparisons. Due to measures (CBCL-Anxious/Depressed, MASC-P and PARS) not meeting satisfactory diagnostic accuracies, only comparisons with the CAIS-P were reported (see Supplemental Appendix 8 for full results). In the complete case analysis, the majority of measures did not differ significantly from the CAIS-P in their diagnostic accuracy for predicting either recovery from all or recovery from primary diagnoses (Table 5). However, DeLong tests on the imputed dataset in favour of the CAIS-P revealed significant differences in diagnostic performance between the CAIS-P and both the CBCL-Anxious/Depressed subscale and the MASC-P in predicting recovery from primary diagnoses, as well as for predicting recovery from all diagnoses (Supplemental Appendix 4).

**Table 5.** Paired comparison of the diagnostic performance of CAIS-P, CBCL-Anxious/Depressed, MASC-P and PARS-Severity for detecting recovery from primary anxiety diagnoses post-treatment.

	Recovery from primary anxiety diagnoses		Recovery from all anxiety diagnoses	
Comparisons	DeLong statistics	Significance levels	DeLong statistics	Significance levels
CAIS-P vs CBCL-Anxious/Depressed	*Z* = −0.812	*p* = 0.417	*Z* = 1.460	*p* = 0.145
CAIS-P vs MASC-P	*Z* = −0.430	*p* = 0.667	*Z* = 1.344	*p* = 0.179
CAIS-P vs PARS	*Z* = 0.418	*p* = 0.676	*Z* = 1.298	*p* = 0.194

CAIS-P: Child Anxiety Impact Scale, Parent Report; CBCL-Anxious/Depressed subscale; MASC-P: Multidimensional Anxiety Scale for Children, Parent Version; PARS: Paediatric Anxiety Rating Scale severity mean score.

Confidence level after Bonferroni correction: *p* = 1 − 0.05/6 = 0.992.

The diagnostic performance of the CAIS-P to predict recovery from primary anxiety diagnoses did not differ significantly according to level of autism traits (as defined by those scoring at the top 30%, those scoring at the middle 40%, and those scoring at the bottom 30% of SRS), number of baseline comorbidities (subgroups are defined by whether autistic children had less than 4, between 4 and 6, or more than 6 baseline diagnoses as assessed by ADIS/ASA), verbal ability (as defined by individual vocabulary scores below sample average, or equal to or above sample average), child sex (males and females) and age group (younger than 10 years old or 10 years old or above; see Supplemental Appendix 8 for full details, see Supplemental Appendix 4 for the imputed results).

In terms of the diagnostic performance of the CAIS-P to predict recovery from all diagnoses, DeLong tests conducted for complete cases did not reveal any significant difference in the pre-specified subgroups in the complete case analysis (Supplemental Appendix 8, see Supplemental Appendix 4 for the imputed results). In the imputed analysis, the findings remain similar, except that particularly high diagnostic accuracy of the CAIS-P was found in girls (AUC = 0.917), which was significantly better than in boys.

## Discussion

Consistent with previous literature with non-autistic populations, we found evidence of acceptable internal consistency and convergent validity for measures of the impact and symptoms of anxiety problems (CAIS-P, CBCL anxious/depressed subscale, MASC-P and PARS) among autistic children. The CAIS-P was the only questionnaire measure to have satisfactory diagnostic accuracy for predicting recovery from all anxiety diagnoses (using the total score), including both *DSM* (American Psychiatric Association, 2013) and other impairing fears that commonly arise in autistic youth.

With regard to the diagnostic accuracy of anxiety symptom and impact measures, our findings indicated both similarities and differences in analyses of data from autistic children compared to previous reports based on non-autistic children (Evans et al., 2017). Similar to Evans et al (2017), we found that the CAIS-P was significantly better than the anxiety symptom measures at predicting full recovery post-treatment (in the imputed datasets), at a level comparable to that reported in non-autistic children. This is likely to be because anxiety impacts and interference are a crucial part of anxiety diagnoses, but are not typically included in conventional anxiety symptom measures. However, none of the measures used here were satisfactorily able to predict recovery from *primary* anxiety diagnoses post-treatment in the context of autism, in contrast to the findings from Evans et al (2017), perhaps reflecting the extensive occurrence of anxiety disorders in this sample and the fact that remission of primary anxiety diagnoses alone (as opposed to multiple or all anxiety diagnoses) may be insufficient to substantially reduce the impact of anxiety among autistic children.

In terms of structural validity, we found poor fit to the original three-factor model with the current CAIS-P data, despite this being well supported in non-autistic populations (cf. Etkin et al., 2024). Unsurprisingly on that basis, we found no evidence of construct validity of the specific CAIS-P subscale that assesses social impact. This finding may be due to anxiety problems affecting autistic children across contexts in different ways. Indeed, Neilson and Bond (2023) highlighted different experiences of anxiety problems among autistic children and young people, with more unaddressed anxiety problems at school, compared to at home or in the community. The poor structural validity could also be because the constructs measured by the CAIS-P subscales are latently confounded by the impact of core autistic characteristics. For example, when filling in a questionnaire like the CAIS-P, a parent might not be able to disentangle whether, for example, a child’s difficulties with developing friendships reflect social communication differences, underlying anxiety, or both. In light of these findings, we recommend caution when interpreting outcomes derived from the CAIS-P. Our analyses highlight the needs for future research to examine factors that may compromise the validity of its subscales. Furthermore, it would be of clinical importance to develop effective prompts for CAIS-P to assist parents in accurately differentiating between impacts of anxiety and of autism when responding to such measures.

The exploratory findings suggest that, when using imputed data, there was no significant difference in the diagnostic accuracy of the CAIS-P in detecting treatment outcomes across subgroups based on different levels of autistic traits, number of baseline comorbidities, verbal comprehension abilities and age. In terms of sex, the results from the imputed datasets suggest that the CAIS-P may have particular potential to detect change in anxiety disorder status in girls. This was not the case for the other symptom measures. This is an important finding given the inconsistent findings in gender differences in anxiety problems (Vasa & Mazurek, 2015) and camouflaging behaviours in autistic youth (Hull et al., 2020). Camouflaging behaviours had significant implications on the misrepresentations and under-recognition of parent- or self-reported anxiety in the context of autism and related challenges in autistic girls (Hull et al., 2020). This therefore highlighted the needs for future studies to fully investigate into the exploratory findings of potentially better diagnostic accuracy of CAIS-P we found in autistic girls, to clarify whether such differences could be generalised, and thus have the potential to overcome the effects of camouflaging. However, it should be noted that outcomes of most subgroup analyses were below satisfactory cutoffs in the complete case analysis, potentially linked to the pattern of missing data. Notably, those with incomplete CAIS-P data were more likely to have recovered from primary or all diagnoses post treatment, as assessed by ADIS/ASA.

Study limitations merit mention: (1) Autistic children’s reports of anxiety problems and post-diagnostic recovery were not examined. It would be crucial for future studies to replicate similar analyses on child-report outcomes to understand whether our current findings can be generalised to autistic children’s subjective experience, especially given the well-reported parent–child disagreement on anxiety outcomes in this population (Kalvin et al., 2020; Weston et al., 2016). (2) No direct comparison was conducted to investigate whether the CAIS-P and our selected symptom measures have comparable diagnostic accuracy in predicting treatment outcomes for autistic and non-autistic children. This limitation is tied to the fact that the anxiety assessments that are currently used were originally developed with non-autistic samples. (3) We were not able to draw conclusions on the diagnostic performance of the CAIS-P or any symptom measures in other scenarios beyond the end of treatment assessments, nor its sensitivity to longitudinal changes. A different study design would be required to establish the ability of the CAIS-P to identify autistic children with anxiety problems from within a community population. While our findings were broadly consistent, it is also important to note that comparisons of these findings with previous findings with non-autistic children (Evans et al., 2017) are limited by the differences in the diagnostic assessment tool used. Here, we used the ADIS/ASA, which was specifically designed to enhance differential diagnoses of anxiety disorders in the context of autism in comparison to the ADIS-C as used in Evans et al (2017). Future studies would usefully directly explore the potential differences of diagnostic accuracy of scale measures in detecting treatment outcomes as assessed by the ADIS-C versus the ADIS/ASA. (4) Future studies would also examine the utility of autism-specific measures of anxiety, for example, the Parent-Rated Anxiety Scale for Youth with Autism Spectrum Disorder (PRAS-ASD) (Scahill et al., 2019), or the Anxiety Scale for Children with Autism Spectrum Disorder (ASC-ASD, Rodgers et al., 2016) to measure treatment outcomes.

## Conclusion

The present findings indicate that the CAIS-P had satisfactory internal consistency and convergent validity in the context of autism, and, although there were some limitations in terms of structural validity, the CAIS-P outperformed symptom measures in terms of its ability to identity recovery from anxiety diagnoses post-treatment. The current findings suggest that the CAIS-P total score could be a useful tool for assessing recovery from anxiety problems within clinical settings.

## Supplemental Material

sj-docx-1-aut-10.1177_13623613251349929 – Supplemental material for How well can commonly used anxiety scales detect treatment outcomes in the context of autism? Supplemental material, sj-docx-1-aut-10.1177_13623613251349929 for How well can commonly used anxiety scales detect treatment outcomes in the context of autism? by Huilin Chen, Jeffrey J Wood, Connor M Kerns, Eric A Storch, Philip C Kendall, Gaia Scerif and Cathy Creswell in Autism

## Notes

1. https://osf.io/qjg6v/registrations.

2. For our secondary exploratory research question, we initially proposed to conduct an ordered logistic regression to look at factors that could affect the diagnostic accuracy of measures for detecting recovery from anxiety diagnostic status. However, the nature of our data did not allow analyses for mutually exclusive subgroups, which would be essential for a logistic regression. We therefore decided to compare diagnostic accuracy across subgroups using the DeLong test (DeLong et al., 1988), with Bonferroni correction to account for multiple comparisons. The DeLong test is a non-parametric method developed specifically to compare the classification performance of ROC (Youngstrom, 2014) and has been frequently adopted in wider clinical research (e.g. Howell et al., 2022; Hu et al., 2020), albeit less so in the field of clinical psychology and psychiatry.

3. Due to extreme imbalance between subgroups (e.g. for race, there was no non-white children who had fully recovered from all anxiety diagnoses in our sample), only two subgroups of age and verbal ability were created, separated by mean. Subgroup analyses of race were aborted due to insufficient variability of data.

4. All results are presented to three decimal places.

## References

[bibr1-13623613251349929] Achenbach T. M. Rescorla L. A. (2001). Manual for the ASEBA school-age forms & profiles. University of Vermont Research Centre for Children, Youth and Families.

[bibr2-13623613251349929] American Psychiatric Association. (2013). Diagnostic and statistical manual of mental disorders (5th ed.).

[bibr3-13623613251349929] Benevides T. W. Shore S. M. Palmer K. Duncan P. Plank A. Andresen M. L. Caplan R. Cook B. Gassner D. Hector B. L. Morgan L. Nebeker L. Purkis Y. Rankowski B. Wittig K. Coughlin S. S. (2020). Listening to the autistic voice: Mental health priorities to guide research and practice in autism from a stakeholder-driven project. Autism, 24(4), 822–833. 32429818 10.1177/1362361320908410 PMC7787673

[bibr4-13623613251349929] Bölte S. Westerwald E. Holtmann M. Freitag C. Poustka F. (2011). Autistic traits and autism spectrum disorders: The clinical validity of two measures presuming a continuum of social communication skills. Journal of Autism and Developmental Disorders, 41, 66–72. 20422277 10.1007/s10803-010-1024-9 PMC3005113

[bibr5-13623613251349929] Bruni T. P. (2014). Test review: Social Responsiveness Scale–Second edition (SRS-2). Journal of Psychoeducational Assessment, 32(4), 365–369.

[bibr6-13623613251349929] Caporino N. E. Sakolsky D. Brodman D. M. McGuire J. F. Piacentini J. Peris T. S. Birmaher B. (2017). Establishing clinical cutoffs for response and remission on the Screen for Child Anxiety Related Emotional Disorders (SCARED). Journal of the American Academy of Child & Adolescent Psychiatry, 56(8), 696–702. 28735699 10.1016/j.jaac.2017.05.018 PMC5546231

[bibr7-13623613251349929] Constantino J. (2002). The Social Responsiveness Scale. Western Psychological Services.

[bibr8-13623613251349929] Creswell C. Nauta M. H. Hudson J. L. March S. Reardon T. Arendt K. Kendall P. C. (2021). Research review: Recommendations for reporting on treatment trials for child and adolescent anxiety disorders: An international consensus statement. Journal of Child Psychology and Psychiatry, 62(3), 255–269. 32683742 10.1111/jcpp.13283

[bibr9-13623613251349929] Creswell C. Waite P. Hudson J. (2020). Practitioner review: Anxiety disorders in children and young people–assessment and treatment. Journal of Child Psychology and Psychiatry, 61(6), 628–643. 31960440 10.1111/jcpp.13186

[bibr10-13623613251349929] DeLong E. R. DeLong D. M. Clarke-Pearson D. L. (1988). Comparing the areas under two or more correlated receiver operating characteristic curves: A nonparametric approach. Biometrics, 44, 837–845. 3203132

[bibr11-13623613251349929] Den Houting J. Adams D. Roberts J. Keen D . (2019). An exploration of autism-specific and non-autism-specific measures of anxiety symptomatology in school-aged autistic children. Clinical Psychologist, 23(3), 237–248.

[bibr12-13623613251349929] DuBois D. Ameis S. H. Lai M. C. Casanova M. F. Desarkar P. (2016). Interoception in autism spectrum disorder: A review. International Journal of Developmental Neuroscience, 52, 104–111. 27269967 10.1016/j.ijdevneu.2016.05.001

[bibr13-13623613251349929] Etkin R. G. Lebowitz E. R. Silverman W. K. (2024). Assessing anxiety-related impairment in children and adolescents. Assessment, 31(1), 94–109. 37840296 10.1177/10731911231194972

[bibr14-13623613251349929] Evans R. Thirlwall K. Cooper P. Creswell C. (2017). Using symptom and interference questionnaires to identify recovery among children with anxiety disorders. Psychological Assessment, 29(7), 835. 27845527 10.1037/pas0000375 PMC5490389

[bibr15-13623613251349929] Glod M. Creswell C. Waite P. Jamieson R. McConachie H. Don South M. Rodgers J. (2017). Comparisons of the factor structure and measurement invariance of the spence children’s anxiety scale—parent version in children with autism spectrum disorder and typically developing anxious children. Journal of Autism and Developmental Disorders, 47, 3834–3846. 28393292 10.1007/s10803-017-3118-0 PMC5676838

[bibr16-13623613251349929] Howell C. R. Zhang L. Yi N. Mehta T. Garvey W. T. Cherrington A. L. (2022). Race versus social determinants of health in COVID-19 hospitalization prediction. American Journal of Preventive Medicine, 63(1), S103–S108. 10.1016/j.amepre.2022.01.034 PMC9212800 35725136

[bibr17-13623613251349929] Hu Q. Whitney H. M. Giger M. L. (2020). A deep learning methodology for improved breast cancer diagnosis using multiparametric MRI. Scientific Reports, 10(1), 10536. 32601367 10.1038/s41598-020-67441-4 PMC7324398

[bibr18-13623613251349929] Hull L. Petrides K. V. Mandy W. (2020). The female autism phenotype and camouflaging: A narrative review. Review Journal of Autism and Developmental Disorders, 7, 306–317.

[bibr19-13623613251349929] Johnco C. J. Salloum A. Lewin A. B. Storch E. A. (2015). Refining clinical judgment of treatment response and symptom remission identification in childhood anxiety using a signal detection analysis on the Pediatric Anxiety Rating Scale. Journal of Child and Adolescent Psychopharmacology, 25(9), 674–683. 26579629 10.1089/cap.2015.0102 PMC4653818

[bibr20-13623613251349929] Kalvin C. B. Marsh C. L. Ibrahim K. Gladstone T. R. Woodward D. Grantz H. Sukhodolsky D. G. (2020). Discrepancies between parent and child ratings of anxiety in children with autism spectrum disorder. Autism Research, 13(1), 93–103. 31643143 10.1002/aur.2220 PMC7062240

[bibr21-13623613251349929] Kerns C. M. Albano A. M. Silverman W. K. (Eds.). (2024). Anxiety and Related Disorders Interview Schedule for DSM-5, Child and Parent Version, with Autism Spectrum Addendum (ADIS/ASA): Parent interview schedule. Oxford University Press.

[bibr22-13623613251349929] Kerns C. M. Kendall P. C. (2012). The presentation and classification of anxiety in autism spectrum disorder. Clinical Psychology: Science and Practice, 19(4), 323.

[bibr23-13623613251349929] Kerns C. M. Kendall P. C. Berry L. Souders M. C. Franklin M. E. Schultz R. T. Herrington J. (2014). Traditional and atypical presentations of anxiety in youth with autism spectrum disorder. Journal of Autism and Developmental Disorders, 44, 2851–2861. 24902932 10.1007/s10803-014-2141-7 PMC5441227

[bibr24-13623613251349929] Kerns C. M. Kendall P. C. Zickgraf H. Franklin M. E. Miller J. Herrington J. (2015). Not to be overshadowed or overlooked: Functional impairments associated with comorbid anxiety disorders in youth with ASD. Behavior Therapy, 46(1), 29–39. 25526833 10.1016/j.beth.2014.03.005

[bibr25-13623613251349929] Kerns C. M. Renno P. Kendall P. C. Wood J. J. Storch E. A. (2017). Anxiety disorders interview schedule–autism addendum: Reliability and validity in children with autism spectrum disorder. Journal of Clinical Child & Adolescent Psychology, 46(1), 88–100. 27925775 10.1080/15374416.2016.1233501 PMC5441235

[bibr26-13623613251349929] Kerns C. M. Winder-Patel B. Iosif A. M. Nordahl C. W. Heath B. Solomon M. Amaral D. G. (2021). Clinically significant anxiety in children with autism spectrum disorder and varied intellectual functioning. Journal of Clinical Child & Adolescent Psychology, 50(6), 780–795. 31971849 10.1080/15374416.2019.1703712 PMC9372909

[bibr27-13623613251349929] Lai M. C. Kassee C. Besney R. Bonato S. Hull L. Mandy W. Ameis S. H. (2019). Prevalence of co-occurring mental health diagnoses in the autism population: A systematic review and meta-analysis. The Lancet Psychiatry, 6(10), 819–829. 31447415 10.1016/S2215-0366(19)30289-5

[bibr28-13623613251349929] Langley A. K. Falk A. Peris T. Wiley J. F. Kendall P. C. Ginsburg G. Birmaher B. March J. Albano A. M. Piacentini J. (2014). The Child Anxiety Impact Scale: Examining parent- and child-reported impairment in child anxiety disorders. Journal of Clinical Child and Adolescent Psychology, 43, 579–591. 23915200 10.1080/15374416.2013.817311 PMC4137893

[bibr29-13623613251349929] Lecavalier L. Wood J. J. Halladay A. K. Jones N. E. Aman M. G. Cook E. H. Scahill L. (2014). Measuring anxiety as a treatment endpoint in youth with autism spectrum disorder. Journal of Autism and Developmental Disorders, 44, 1128–1143. 24158679 10.1007/s10803-013-1974-9 PMC3981870

[bibr30-13623613251349929] Lord C. Risi S. Lambrecht L. Cook E. H. Jr. Leventhal B. L. DiLavore P. C. Pickles A. Rutter M. (2000). The Autism Diagnostic Observation Schedule-Generic: A standard measure of social and communication deficits associated with the spectrum of autism. Journal of Autism and Developmental Disorders, 30(3), 205–223. 11055457

[bibr31-13623613251349929] March J. S. Parker J. D. Sullivan K. Stallings P. Conners C. K. (1997). The Multidimensional Anxiety Scale for Children (MASC): Factor structure, reliability, and validity. Journal of the American Academy of Child & Adolescent Psychiatry, 36(4), 554–565. 9100431 10.1097/00004583-199704000-00019

[bibr32-13623613251349929] Mayes S. D. Calhoun S. L. (2008). WISC-IV and WIAT-II profiles in children with high-functioning autism. Journal of Autism and Developmental Disorders, 38, 428–439. 17610151 10.1007/s10803-007-0410-4

[bibr33-13623613251349929] Neilson C. Bond C. (2023). The experience of anxiety for autistic children and young people: A thematic synthesis review. Research in Autism Spectrum Disorders, 109, 102274.

[bibr34-13623613251349929] Oliveras-Rentas R. E. Kenworthy L. Roberson R. B. Martin A. Wallace G. L. (2012). WISC-IV profile in high-functioning autism spectrum disorders: Impaired processing speed is associated with increased autism communication symptoms and decreased adaptive communication abilities. Journal of Autism and Developmental Disorders, 42, 655–664. 21638108 10.1007/s10803-011-1289-7 PMC3448485

[bibr35-13623613251349929] Palitz S. A. Caporino N. E. McGuire J. F. Piacentini J. Albano A. M. Birmaher B. Kendall P. C. (2018). Defining treatment response and remission in youth anxiety: A signal detection analysis with the multidimensional anxiety scale for children. Journal of the American Academy of Child & Adolescent Psychiatry, 57(6), 418–427. 29859557 10.1016/j.jaac.2018.03.013 PMC5988233

[bibr36-13623613251349929] Palser E. R. Fotopoulou A. Pellicano E. Kilner J. M. (2018). The link between interoceptive processing and anxiety in children diagnosed with autism spectrum disorder: Extending adult findings into a developmental sample. Biological Psychology, 136, 13–21. 29742462 10.1016/j.biopsycho.2018.05.003

[bibr37-13623613251349929] Pandolfi V. Magyar C. I. Dill C. A. (2012). An initial psychometric evaluation of the CBCL 6–18 in a sample of youth with autism spectrum disorders. Research in Autism Spectrum Disorders, 6(1), 96–108. 22059091 10.1016/j.rasd.2011.03.009 PMC3207215

[bibr38-13623613251349929] Research Units on Pediatric Psychopharmacology Anxiety Study Group. (2002). The Pediatric Anxiety Rating Scale (PARS): Development and psychometric properties. Journal of the American Academy of Child & Adolescent Psychiatry, 41(9), 1061–1069. 12218427 10.1097/00004583-200209000-00006

[bibr39-13623613251349929] Rodgers J. Wigham S. McConachie H. Freeston M. Honey E. Parr J. R. (2016). Development of the Anxiety Scale for Children with Autism Spectrum Disorder (ASC-ASD). Autism Research, 9(11), 1205–1215. 26887910 10.1002/aur.1603

[bibr40-13623613251349929] Rynn M. A. Barber J. P. Khalid-Khan S. Siqueland L. Dembiski M. McCarthy K. S. Gallop R. (2006). The psychometric properties of the MASC in a pediatric psychiatric sample. Journal of Anxiety Disorders, 20(2), 139–157. 16464701 10.1016/j.janxdis.2005.01.004

[bibr41-13623613251349929] Scahill L. Lecavalier L. Schultz R. T. Evans A. N. Maddox B. Pritchett J. Edwards M. C. (2019). Development of the parent-rated anxiety scale for youth with autism spectrum disorder. Journal of the American Academy of Child & Adolescent Psychiatry, 58(9), 887–896. 30797036 10.1016/j.jaac.2018.10.016

[bibr42-13623613251349929] Schober P. Boer C. Schwarte L. A. (2018). Correlation coefficients: Appropriate use and interpretation. Anesthesia & Analgesia, 126(5), 1763–1768. 29481436 10.1213/ANE.0000000000002864

[bibr43-13623613251349929] Schopler E. Van Bourgondien M. E. Wellman G. J. Love S. R. (2010). Childhood Autism Rating Scale™ (2nd ed.). WPS Publishing.

[bibr44-13623613251349929] Stekhoven D. J. Bühlmann P. (2012). MissForest – Non-parametric missing value imputation for mixed-type data. Bioinformatics, 28(1), 112–118. 22039212 10.1093/bioinformatics/btr597

[bibr45-13623613251349929] Storch E. A. Wood J. J. Ehrenreich-May J. Jones A. M. Park J. M. Lewin A. B. Murphy T. K. (2012). Convergent and discriminant validity and reliability of the pediatric anxiety rating scale in youth with autism spectrum disorders. Journal of Autism and Developmental Disorders, 42, 2374–2382. 22395820 10.1007/s10803-012-1489-9

[bibr46-13623613251349929] Thaler N. S. Kazemi E. Wood J. J. (2010). Measuring anxiety in youth with learning disabilities: Reliability and validity of the Multidimensional Anxiety Scale for Children (MASC). Child Psychiatry & Human Development, 41, 501–514. 20405202 10.1007/s10578-010-0182-5 PMC2917554

[bibr47-13623613251349929] Van Steensel F. J. Bögels S. M. Perrin S . (2011). Anxiety disorders in children and adolescents with autistic spectrum disorders: A meta-analysis. Clinical Child and Family Psychology Review, 14, 302–317. 21735077 10.1007/s10567-011-0097-0 PMC3162631

[bibr48-13623613251349929] Vasa R. A. Mazurek M. O. (2015). An update on anxiety in youth with autism spectrum disorders. Current Opinion in Psychiatry, 28(2), 83–90. 25602249 10.1097/YCO.0000000000000133 PMC5764108

[bibr49-13623613251349929] Villabø M. Gere M. Torgersen S. March J. S. Kendall P. C. (2012). Diagnostic efficiency of the child and parent versions of the Multidimensional Anxiety Scale for Children. Journal of Clinical Child & Adolescent Psychology, 41(1), 75–85. 22233247 10.1080/15374416.2012.632350

[bibr50-13623613251349929] Wechsler D. (2003). Wechsler Intelligence Scale for Children (4th ed.). PsychCorp.

[bibr51-13623613251349929] Weston L. Hodgekins J. Langdon P. E. (2016). Effectiveness of cognitive behavioural therapy with people who have autistic spectrum disorders: A systematic review and meta-analysis. Clinical Psychology Review, 49, 41–54. 27592496 10.1016/j.cpr.2016.08.001

[bibr52-13623613251349929] White S. W. Lerner M. D. McLeod B. D. Wood J. J. Ginsburg G. S. Kerns C. Compton S. (2015). Anxiety in youth with and without autism spectrum disorder: Examination of factorial equivalence. Behavior Therapy, 46(1), 40–53. 25526834 10.1016/j.beth.2014.05.005 PMC4273846

[bibr53-13623613251349929] Wood J. J. Kendall P. C. Wood K. S. Kerns C. M. Seltzer M. Small B. J. Storch E. A. (2020). Cognitive behavioral treatments for anxiety in children with autism spectrum disorder: A randomized clinical trial. JAMA Psychiatry, 77(5), 474–483. 31755906 10.1001/jamapsychiatry.2019.4160 PMC6902190

[bibr54-13623613251349929] Youngstrom E. A. (2014). A primer on receiver operating characteristic analysis and diagnostic efficiency statistics for pediatric psychology: We are ready to ROC. Journal of Pediatric Psychology, 39(2), 204–221. 23965298 10.1093/jpepsy/jst062 PMC3936258

[bibr55-13623613251349929] Zablotsky B. Pringle B. A. Colpe L. J. Kogan M. D. Rice C. Blumberg S. J. (2015). Service and treatment use among children diagnosed with autism spectrum disorders. Journal of Developmental & Behavioral Pediatrics, 36(2), 98–105. 25650952 10.1097/DBP.0000000000000127 PMC4646056

