# Supplementary material for: How well can commonly used anxiety scales detect treatment outcomes in the context of autism?
Source: Autism. 2025 Jul 5;29(12):2991–3001. doi: 10.1177/13623613251349929 (PMC12360284; doi:10.1177/13623613251349929)
Supplement: sj-docx-1-aut-10.1177_13623613251349929 – Supplemental material for How well can commonly used anxiety scales detect treatment outcomes in the context of autism? [file sj-docx-1-aut-10.1177_13623613251349929.docx]

Supplementary Materials

Appendix 1. Sample baseline diagnostic characteristic

- 1. Frequencies of pre-treatment primary anxiety diagnoses as diagnosed by ADIS/ASA

| Eligibility | Diagnoses | Frequencies |
| --- | --- | --- |
| Eligible participants (*N* =167) | Separation Anxiety Disorder | 12 |
|  | Social Phobia | 32 |
|  | Atypical Social Fear | 3 |
|  | Specific Phobia | 17 |
|  | Atypical Specific Fear | 2 |
|  | Generalized Anxiety Disorder | 69 |
|  | Special Interest Fear | 2 |
|  | Fear of Change | 5 |
|  | Negative Reaction to Change | 1 |
| Screened participants  (*N* =46) | Social Phobia | 7 |
|  | Atypical Social Fear | 2 |
|  | Specific Phobia | 1 |
|  | Atypical Specific Fear | 1 |
|  | Generalized Anxiety Disorder | 10 |
|  | Fear of Change | 5 |
|  | Negative Reaction to Change | 1 |

- 1. Average number of pre-treatment comorbid diagnoses

|  | Average number of pre-treatment comorbid diagnoses  *M* (*SD*) |
| --- | --- |
| Eligible participants | 5.53 (2.14) |
| Screened participants | 4.83 (2.04) |

- 1. Frequencies of anxiety diagnoses in the eligible sample pre-treatment

| DSM or ASA specific diagnosis | Diagnoses | Frequencies |
| --- | --- | --- |
| DSM Diagnoses | Separation Anxiety Disorder | 61 |
|  | Social Phobia | 102 |
|  | Specific Phobia* | 107 |
|  | Generalized Anxiety Disorder | 149 |
| ASA-specific diagnosis | Special Interest Fear | 6 |
|  | Fear of Change | 56 |
|  | Negative Reaction to Change | 23 |
|  | Atypical Anxiety | 85 |

**Note*: Of those children with a baseline specific phobia diagnosis, 42 had only 1 specific phobia diagnoses and 65 had multiple specific phobia diagnoses.

1.3. Number of participants with only DSM or ASA anxiety diagnoses, or both, in the eligible sample pre-treatment

|  | Frequencies |
| --- | --- |
| Participants with only DSM or ASA anxiety Diagnoses | 79 |
| Participant with both DSM and ASA anxiety Diagnoses | 84 |

Appendix 2. Assumption Tests

| Tests of Normality | | | | | | |
| --- | --- | --- | --- | --- | --- | --- |
|  | Kolmogorov-Smirnova | | | Shapiro-Wilk | | |
|  | Statistic | df | Sig. | Statistic | df | Sig. |
| Pre Treatment CAIS-P total scores | 0.076 | 116 | 0.094 | 0.993 | 116 | 0.825 |
| Post Treatment CAIS-P total scores | 0.112 | 116 | 0.001* | 0.939 | 116 | <.001* |
| Pre Treatment CBCL anxious/depressed scores | 0.099 | 116 | 0.007* | 0.974 | 116 | 0.026* |
| Post Treatment CBCL anxious/depressed scores | 0.086 | 116 | 0.034* | 0.967 | 116 | 0.006* |
| Pre Treatment MASC-P total scores | 0.065 | 116 | .200 | 0.981 | 116 | 0.1 |
| Post Treatment MASC-P total score | 0.062 | 116 | .200 | 0.991 | 116 | 0.683 |
| Pre Treatment PARS severity mean scores | 0.107 | 116 | 0.002* | 0.967 | 116 | 0.005* |
| Post Treatment PARS severity mean scores | 0.064 | 116 | .200 | 0.981 | 116 | 0.102 |
| Pre Treatment CBCL social competence mean scores | 0.117 | 116 | <.001* | 0.964 | 116 | 0.003* |
| Post treatment CBCL social competence mean scores | 0.112 | 116 | 0.001* | 0.961 | 116 | 0.002* |

*p<0.05

*Note.* CAIS-P: Child Anxiety Impact Scale, Parent Report; CBCL-Anxious/Depressed subscale; MASC-P: Multidimensional Anxiety Scale for Children, Parent Version; PARS: Pediatric Anxiety Rating Scale severity mean score

| Bartlett's Test of Sphericity | | | |
| --- | --- | --- | --- |
|  | Approx. Chi-Square | df | Significance |
| Pre Treatment CAIS-P item scores | 1904.598 | 351 | <0.001* |
| Post Treatment CAIS-P item scores | 1592.015 | 351 | <0.001* |

*p<0.05

| Grubb's Test of Outliers | | | |
| --- | --- | --- | --- |
|  | G statistic | U | Significance |
| Pre Treatment CAIS-P total scores | 2.744 | 0.962 | 0.565 |
| Post Treatment CAIS-P total scores | 2.926 | 0.935 | 0.199 |
| Pre Treatment CBCL anxious/depressed scores | 2.611 | 0.966 | 0.863 |
| Post Treatment CBCL anxious/depressed scores | 2.847 | 0.939 | 0.263 |
| Pre Treatment CBCL social competence mean scores | 2.240 | 0.976 | 1.00 |
| Post Treatment CBCL social competence mean scores | 3.748 | 0.895 | 0.008* |
| Pre Treatment MASC-P scores | 3.132 | 0.950 | 0.153 |
| Post Treatment MASC-P scores | 3.10463 | 0.92698 | 0.1066 |
| Pre Treatment PARS severity mean scores | 4.01578 | 0.92172 | 0.00443* |
| Post Treatment PARS severity mean scores | 2.84975 | 0.94241 | 0.2781 |

*p<0.05, for post treatment CBCL social competence mean scores, highest value 3.6 is an outlier and for pre treatment PARS severity mean score, lowest value 1.41 is an outlier.

| Measures | Pre-treatment Cronbach’s alpha | Post-treatment Cronbach’s alpha |
| --- | --- | --- |
| CAIS-P | 0.854 | 0.884 |
| CBCL | 0.943 | 0.951 |
| MASC-P | 0.863 | 0.870 |

*Note.* CAIS-P: Child Anxiety Impact Scale, Parent Report; CBCL-Anxious/Depressed subscale; MASC-P: Multidimensional Anxiety Scale for Children, Parent Version

Appendix 3. Data Missingness Examination

| Little's MCAR test | | |
| --- | --- | --- |
| Chi square | df | Significance |
| 224.243 | 46 | <0.001 |

| **Descriptive Statistics** | | | | | | |
| --- | --- | --- | --- | --- | --- | --- |
| Cases with missing post treatment CAIS-P data versus those with complete data | | N | Minimum | Maximum | Mean | s.d. |
| Complete CAIS-P data | Post treatment CBCL-P Social competence mean scores | 133 | 1.00 | 3.60 | 2.022 | .426 |
|  | Number of baseline comorbid anxiety disorders | 133 | 1.00 | 10.00 | 5.459 | 2.183 |
|  | Full-scale IQ | 133 | 1.00 | 3.00 | 2.023 | .812 |
|  | Vocabulary comprehension | 127 | 1.00 | 3.00 | 1.921 | .803 |
|  | Autism traits | 127 | 1.00 | 3.00 | 1.930 | .768 |
|  | Post treatment CBCL anxious/depressed scores | 132 | 1 | 20 | 7.890 | 4.149 |
|  | Recovery from primary diagnoses | 133 | 1.00 | 2.00 | 1.895 | .308 |
|  | Recover from all anxiety diagnoses | 133 | 1.00 | 2.00 | 1.955 | .208 |
|  | Gender (1 boys 2 girls) | 133 | 1.00 | 2.00 | 1.218 | .4145 |
|  | Post treatment mean of all 7 pars severity items | 131 | .00 | 4.29 | 2.270 | .793 |
| Cases with missing CAIS-P data | Post treatment CBCL-P Social competence mean scores | 3 | 1.83 | 2.00 | 1.944 | .096 |
|  | Number of baseline comorbid anxiety disorders | 75 | 1.00 | 10.00 | 5.2800 | 2.044 |
|  | Full-scale IQ | 80 | 1.00 | 3.00 | 1.900 | .836 |
|  | Vocabulary comprehension | 70 | 1.00 | 3.00 | 1.771 | .765 |
|  | Autism traits | 73 | 1.00 | 3.00 | 2.110 | .774 |
|  | Post treatment CBCL anxious/depressed scores | 3 | 10 | 16 | 13.67 | 3.215 |
|  | Recovery from primary diagnoses | 75 | 1.00 | 2.00 | 1.120 | .327 |
|  | Recover from all anxiety diagnoses | 75 | 1.00 | 2.00 | 1.133 | .342 |
|  | Gender (1 boys 2 girls) | 79 | 1.00 | 2.00 | 1.203 | .404 |
|  | Post treatment mean of all 7 pars severity items | 12 | 2.43 | 4.14 | 3.333 | .539 |

*Note.* CAIS-P: Child Anxiety Impact Scale, Parent Report; CBCL-Anxious/Depressed subscale; MASC-P: Multidimensional Anxiety Scale for Children, Parent Version; PARS: Pediatric Anxiety Rating Scale severity mean score

| **Hypothesis Test Summary** | | | | |
| --- | --- | --- | --- | --- |
|  | Null Hypothesis | Test | Sig.^a,b^ | Decision |
| 1 | The distribution of Post treatment CBCL-P Social competence mean scores is the same across categories of cases with missing or complete post treatment CAIS-P data | Independent-Samples Mann-Whitney U Test | .823 | Retain the null hypothesis. |
| 2 | The distribution of Post treatment CBCL-P Social competence mean scores is the same across categories of cases with missing or complete post treatment CAIS-P data. | Independent-Samples Kolmogorov-Smirnov Test | .654 | Retain the null hypothesis. |
| 3 | The distribution of Autism traits is the same across categories of cases with missing or complete post treatment CAIS-P data. | Independent-Samples Mann-Whitney U Test | .112 | Retain the null hypothesis. |
| 4 | The distribution of Autism traits is the same across categories of cases with missing or complete post treatment CAIS-P data. | Independent-Samples Kolmogorov-Smirnov Test | .783 | Retain the null hypothesis. |
| 5 | The distribution of Vocabulary comprehension is the same across categories of cases with missing or complete post treatment CAIS-P data. | Independent-Samples Mann-Whitney U Test | .212 | Retain the null hypothesis. |
| 6 | The distribution of Vocabulary comprehension is the same across categories of cases with missing or complete post treatment CAIS-P data. | Independent-Samples Kolmogorov-Smirnov Test | .912 | Retain the null hypothesis. |
| 7 | The distribution of Full-scale IQ is the same across categories of cases with missing or complete post treatment CAIS-P data. | Independent-Samples Mann-Whitney U Test | .288 | Retain the null hypothesis. |
| 8 | The distribution of Full-scale IQ is the same across categories of cases with missing or complete post treatment CAIS-P data. | Independent-Samples Kolmogorov-Smirnov Test | .871 | Retain the null hypothesis. |
| 9 | The distribution of Number of baseline comorbid anxiety disorders is the same across categories of cases with missing or complete post treatment CAIS-P data. | Independent-Samples Mann-Whitney U Test | .587 | Retain the null hypothesis. |
| 10 | The distribution of Number of baseline comorbid anxiety disorders is the same across categories of cases with missing or complete post treatment CAIS-P data. | Independent-Samples Kolmogorov-Smirnov Test | .993 | Retain the null hypothesis. |
| 11 | The distribution of Post treatment CBCL anxious/depressed scores is the same across categories of cases with missing or complete post treatment CAIS-P data. | Independent-Samples Mann-Whitney U Test | .022 | Reject the null hypothesis. |
| 12 | The distribution of Post treatment CBCL anxious/depressed scores is the same across categories of cases with missing or complete post treatment CAIS-P data. | Independent-Samples Kolmogorov-Smirnov Test | .139 | Retain the null hypothesis. |
| 13 | The distribution of Post treatment MASC-P is the same across categories of cases with missing or complete post treatment CAIS-P data. | Independent-Samples Mann-Whitney U Test | .607 | Retain the null hypothesis. |
| 14 | The distribution of Post treatment MASC-P is the same across categories of cases with missing or complete post treatment CAIS-P data. | Independent-Samples Kolmogorov-Smirnov Test | .895 | Retain the null hypothesis. |
| 15 | The distribution of posttreatment mean of all 7 pars severity items is the same across categories of cases with missing or complete post treatment CAIS-P data. | Independent-Samples Mann-Whitney U Test | <.001 | Reject the null hypothesis. |
| 16 | The distribution of posttreatment mean of all 7 pars severity items is the same across categories of cases with missing or complete post treatment CAIS-P data. | Independent-Samples Kolmogorov-Smirnov Test | <.001 | Reject the null hypothesis. |
| a. The significance level is .050. | | | | |

*Note.* CAIS-P: Child Anxiety Impact Scale, Parent Report; CBCL-Anxious/Depressed subscale; MASC-P: Multidimensional Anxiety Scale for Children, Parent Version; PARS: Pediatric Anxiety Rating Scale severity mean score

| Recovery from all anxiety diagnoses between completers and non-completers of CAIS-P  Chi-Square Tests | | | | | |
| --- | --- | --- | --- | --- | --- |
|  | Value | df | Asymptotic Significance (2-sided) | Exact Sig. (2-sided) | Exact Sig. (1-sided) |
| Pearson Chi-Square | 80.224^a^ | 1 | <.001* |  |  |
| Continuity Correction^b^ | 75.803 | 1 | <.001* |  |  |
| Likelihood Ratio | 67.161 | 1 | <.001* |  |  |
| Fisher's Exact Test |  |  |  | <.001 | <.001 |
| Linear-by-Linear Association | 79.744 | 1 | <.001* |  |  |

*p<0.001

| Recovery from primary anxiety diagnoses between completers and non-completers of CAIS-P  Chi-Square Tests | | | | | |
| --- | --- | --- | --- | --- | --- |
|  | Value | df | Asymptotic Significance (2-sided) | Exact Sig. (2-sided) | Exact Sig. (1-sided) |
| Pearson Chi-Square | 60.048^a^ | 1 | <.001* |  |  |
| Continuity Correction^b^ | 56.579 | 1 | <.001* |  |  |
| Likelihood Ratio | 52.726 | 1 | <.001* |  |  |
| Fisher's Exact Test |  |  |  | <.001* | <.001* |
| Linear-by-Linear Association | 59.688 | 1 | <.001* |  |  |

*p<0.01

Appendix 4. Imputed Results

4.1. Imputed results for diagnostic accuracy of predicting recovery from primary anxiety diagnoses

| Measures | Imputed Data Diagnostic accuracy |
| --- | --- |
| CAIS-P | 0.676 |
| CBCL-Anxious/Depressed | 0.498 |
| MASC-P | 0.522 |
| PARS-severity mean score | 0.655 |

*Note.* CAIS-P: Child Anxiety Impact Scale, Parent Report; CBCL-Anxious/Depressed subscale; MASC-P: Multidimensional Anxiety Scale for Children, Parent Version; PARS: Pediatric Anxiety Rating Scale severity mean score

4.2. Imputed results for paired comparisons of diagnostic accuracy of predicting recovery from primary anxiety diagnoses across measures

| Comparisons | DeLong statistics | p values |
| --- | --- | --- |
| CAIS-P vs CBCL-Anxious/Depressed | Z =4.110 | p<0.001* |
| CAIS-P vs MASC-P | Z = 3.582 | p<0.001* |
| CAIS-P vs PARS | Z = 0.507 | p=0.612 |
| CBCL-Anxious/Depressed vs MASC-P | Z = -0.579 | p=0.563 |
| CBCL-Anxious/Depressed vs PARS | Z = -3.601 | p<0.001* |
| MASC-P vs PARS | Z = -2.928 | p=0.003* |

*p<0.00833, significance level after Bonferroni correction

*Note.* CAIS-P: Child Anxiety Impact Scale, Parent Report; CBCL-Anxious/Depressed subscale; MASC-P: Multidimensional Anxiety Scale for Children, Parent Version; PARS: Pediatric Anxiety Rating Scale severity mean score

4.3. Imputed results for subgroup comparisons of diagnostic accuracy of predicting recovery from primary anxiety diagnoses

4.3.1. Comparisons of diagnostic accuracy across different levels of autism traits

| Measures | Different levels of autism traits | Imputed Data  Diagnostic Accuracy |
| --- | --- | --- |
| CAIS-P | Mild | 0.756 |
|  | Moderate | 0.668 |
|  | Severe | 0.615 |
| CBCL-Anxious/Depressed | Mild | 0.589 |
|  | Moderate | 0.435 |
|  | Severe | 0.496 |
| MASC-P | Mild | 0.565 |
|  | Moderate | 0.587 |
|  | Severe | 0.456 |
| PARS-severity mean score | Mild | 0.689 |
|  | Moderate | 0.687 |
|  | Severe | 0.593 |

Mild: SRS scores at or below the bottom 30% of the sample; Moderate: SRS scores between the middle 40% of the sample; severe: SRS scores at or above the top 30% of the sample

*Note.* CAIS-P: Child Anxiety Impact Scale, Parent Report; CBCL-Anxious/Depressed subscale; MASC-P: Multidimensional Anxiety Scale for Children, Parent Version; PARS: Pediatric Anxiety Rating Scale severity mean score

| **DeLong tests on imputed data** | | | |
| --- | --- | --- | --- |
| Measures | sub-group analysis | DeLong statistics | p values |
| CAIS-P | Mild versus moderate | D = 0.839 | p=0.404 |
|  | Mild versus severe | D = -1.190 | p=0.238 |
|  | Moderate versus severe | D = 0.406 | p=0.686 |
| CBCL-Anxious/Depressed | Mild versus moderate | D = 1.310 | p=0.193 |
|  | Mild versus severe | D = 1.190 | p=0.446 |
|  | Moderate versus severe | D = -0.504 | p=0.615 |
| MASC-P | Mild versus moderate | D = -0.181 | p=0.856 |
|  | Mild versus severe | D = 0.909 | p=0.366 |
|  | Moderate versus severe | D = 1.081 | p=0.282 |
| PARS-severity mean score | Mild versus moderate | D = 0.022 | p= 0.983 |
|  | Mild versus severe | D = 0.821 | p=0.414 |
|  | Moderate versus severe | D = 0.774 | p=0.441 |

Mild: SRS scores at or below the bottom 30% of the sample; Moderate: SRS scores between the middle 40% of the sample; severe: SRS scores at or above the top 30% of the sample

*Note.* CAIS-P: Child Anxiety Impact Scale, Parent Report; CBCL-Anxious/Depressed subscale; MASC-P: Multidimensional Anxiety Scale for Children, Parent Version; PARS: Pediatric Anxiety Rating Scale severity mean score

4.3.2. Comparisons of diagnostic accuracy across different numbers of baseline comorbidities

| Measures | Different numbers of baseline comorbidities grouping | Imputed Data  Diagnostic accuracy |
| --- | --- | --- |
| CAIS-P | <4 | 0.554 |
|  | [4,6) | 0.609 |
|  | >=6 | 0.714 |
| CBCL-Anxious/Depressed | <4 | 0.571 |
|  | [4,6) | 0.522 |
|  | >=6 | 0.513 |
| MASC-P | <4 | 0.658 |
|  | [4,6) | 0.544 |
|  | >=6 | 0.537 |
| PARS-severity mean score | <4 | 0.604 |
|  | [4,6) | 0.629 |
|  | >=6 | 0.604 |

*Note.* CAIS-P: Child Anxiety Impact Scale, Parent Report; CBCL-Anxious/Depressed subscale; MASC-P: Multidimensional Anxiety Scale for Children, Parent Version; PARS: Pediatric Anxiety Rating Scale severity mean score

| **DeLong test on imputed data** | | | |
| --- | --- | --- | --- |
|  | sub-group analysis | DeLong statistics | p values |
| CAIS-P | less than 4 baseline comorbid anxiety Dx versus 4-6 baseline comorbid anxiety Dx | D = -0.276 | p=0.784 |
|  | less than 4 baseline comorbid anxiety Dx versus more than 6 baseline comorbid anxiety Dx | D = -0.849 | p=0.401 |
|  | 4-6 baseline comorbid anxiety Dx versus more than 6 baseline comorbid anxiety Dx | D = -1.015 | p=0.313 |
| CBCL-Anxious/Depressed | less than 4 baseline comorbid anxiety Dx versus 4-6 baseline comorbid anxiety Dx | D = 0.317 | p=0.753 |
|  | less than 4 baseline comorbid anxiety Dx versus more than 6 baseline comorbid anxiety Dx | D = 0.407 | p=0.686 |
|  | 4-6 baseline comorbid anxiety Dx versus more than 6 baseline comorbid anxiety Dx | D = 0.089 | p=0.930 |
| MASC-P | less than 4 baseline comorbid anxiety Dx versus 4-6 baseline comorbid anxiety Dx | D = 0.651830142 | p=0.5173 |
|  | less than 4 baseline comorbid anxiety Dx versus more than 6 baseline comorbid anxiety Dx | D = 0.732 | p=0.468 |
|  | 4-6 baseline comorbid anxiety Dx versus more than 6 baseline comorbid anxiety Dx | D = 0.064 | p=0.280 |
| PARS-severity mean score | less than 4 baseline comorbid anxiety Dx versus 4-6 baseline comorbid anxiety Dx | D = -0.169 | p=0.867 |
|  | less than 4 baseline comorbid anxiety Dx versus more than 6 baseline comorbid anxiety Dx | D = -0.416 | p=0.679 |
|  | 4-6 baseline comorbid anxiety Dx versus more than 6 baseline comorbid anxiety Dx | D = -0.305 | p=0.761 |

*Note.* CAIS-P: Child Anxiety Impact Scale, Parent Report; CBCL-Anxious/Depressed subscale; MASC-P: Multidimensional Anxiety Scale for Children, Parent Version; PARS: Pediatric Anxiety Rating Scale severity mean score; Dx: diagnoses

4.3.3. Comparisons of diagnostic accuracy across different levels of verbal abilities

| Measures | Different levels of vocabulary comprehension | Imputed data  diagnostic accuracy |
| --- | --- | --- |
| CAIS-P | Bottom 30% of vocabulary comprehension | 0.631 |
|  | Middle 40% of vocabulary comprehension | 0.614 |
|  | Top 30% of vocabulary comprehension | 0.823 |
| CBCL-Anxious/Depressed | Bottom 30% of vocabulary comprehension | 0.541 |
|  | Middle 40% of vocabulary comprehension | 0.558 |
|  | Top 30% of vocabulary comprehension | 0.568 |
| MASC-P | Bottom 30% of vocabulary comprehension | 0.593 |
|  | Middle 40% of vocabulary comprehension | 0.574 |
|  | Top 30% of vocabulary comprehension | 0.591 |
| PARS-severity mean score | Bottom 30% of vocabulary comprehension | 0.727 |
|  | Middle 40% of vocabulary comprehension | 0.610 |
|  | Top 30% of vocabulary comprehension | 0.604 |

*Note.* CAIS-P: Child Anxiety Impact Scale, Parent Report; CBCL-Anxious/Depressed subscale; MASC-P: Multidimensional Anxiety Scale for Children, Parent Version; PARS: Pediatric Anxiety Rating Scale severity mean score

| **DeLong tests on imputed data** | | | |
| --- | --- | --- | --- |
| Measures | sub-group analysis | DeLong statistics | p values |
| CAIS-P | Low versus Medium | D =0.142 | p=0.887 |
|  | Low versus High | D = -1.817 | p=0.072 |
|  | Medium versus High | D = -1.973 | **p=0.051 (trend)** |
| CBCL-Anxious/Depressed | Low versus Medium | D = - 0.154 | p=0.878 |
|  | Low versus High | D = -0.232 | p=0.817 |
|  | Medium versus High | D = -0.083 | p=0.934 |
| MASC-P | Low versus Medium | D = 0.161 | p=0.873 |
|  | Low versus High | D =0.014 | p=0.989 |
|  | Medium versus High | D = -0.134 | p=0.894 |
| PARS-severity mean score | Low versus Medium | D = 1.097 | p=0.275 |
|  | Low versus High | D = 1.040 | p=0.301 |
|  | Medium versus High | D = 0.055 | p=0.956 |

Low: WISC-vocabulary subscale scores at or below the bottom 30%; Medium: WISC-vocabulary subscale scores between the middle 40%; High: WISC-vocabulary subscale scores at or above the top 30%

*Note.* CAIS-P: Child Anxiety Impact Scale, Parent Report; CBCL-Anxious/Depressed subscale; MASC-P: Multidimensional Anxiety Scale for Children, Parent Version; PARS: Pediatric Anxiety Rating Scale severity mean score

4.3.4 Comparisons of diagnostic accuracy across different sexes

| Measures | Sex | Imputed data diagnostic accuracy |
| --- | --- | --- |
| CAIS-P | Male | 0.632 |
|  | Female | 0.791 |
| CBCL-Anxious/Depressed | Male | 0.493 |
|  | Female | 0.511 |
| MASC-P | Male | 0.483 |
|  | Female | 0.564 |
| PARS-severity mean score | Male | 0.636 |
|  | Female | 0.738 |

*Note.* CAIS-P: Child Anxiety Impact Scale, Parent Report; CBCL-Anxious/Depressed subscale; MASC-P: Multidimensional Anxiety Scale for Children, Parent Version; PARS: Pediatric Anxiety Rating Scale severity mean score

| **DeLong tests on imputed data** | | | |
| --- | --- | --- | --- |
| Measures | sub-group analysis | DeLong statistics | p values |
| CAIS-P | Male versus female | D = -1.134 | p=0.263 |
| CBCL-Anxious/Depressed | Male versus female | D =-0.145 | p=0.885 |
| MASC-P | Male versus female | D = -0.586 | p=0.561 |
| PARS-severity mean score | Male versus female | D =-1.026 | p=0.309 |

*Note.* CAIS-P: Child Anxiety Impact Scale, Parent Report; CBCL-Anxious/Depressed subscale; MASC-P: Multidimensional Anxiety Scale for Children, Parent Version; PARS: Pediatric Anxiety Rating Scale severity mean score

4.3.5. Comparisons of diagnostic accuracy for those younger than 10 years old, or 10 years old and above

We chose 10 years old as a cutoff for age because it was the closest year to both the upper and lower bound of 30 percentile and also the median.

| Measures | Age | Imputed Data Diagnostic Accuracy |
| --- | --- | --- |
| CAIS-P | <10 | 0.664 |
|  | >=10 | 0.696 |
| CBCL-Anxious/Depressed | <10 | 0.447 |
|  | >=10 | 0.558 |
| MASC-P | <10 | 0.488 |
|  | >=10 | 0.455 |
| PARS-severity mean score | <10 | 0.616 |
|  | >=10 | 0.686 |

*Note.* CAIS-P: Child Anxiety Impact Scale, Parent Report; CBCL-Anxious/Depressed subscale; MASC-P: Multidimensional Anxiety Scale for Children, Parent Version; PARS: Pediatric Anxiety Rating Scale severity mean score

| **DeLong test on imputed data** | | | |
| --- | --- | --- | --- |
| Measures | sub-group analysis | DeLong statistics | p values |
| CAIS-P | <10 years versus >=10 years | D =0.351 | p=0.726 |
| CBCL-Anxious/Depressed | <10 years versus >=10 years | D = -1.186 | p=0.238 |
| MASC-P | <10 years versus >=10 years | D = 0.356 | p=0.723 |
| PARS-severity mean score | <10 years versus >=10 years | D =0.785 | p=0.434 |

*Note.* CAIS-P: Child Anxiety Impact Scale, Parent Report; CBCL-Anxious/Depressed subscale; MASC-P: Multidimensional Anxiety Scale for Children, Parent Version; PARS: Pediatric Anxiety Rating Scale severity mean score

4.4. Imputed results for subgroup comparisons of diagnostic accuracy of predicting recovery from all anxiety diagnoses

| Measures | Imputed Data  Diagnostic accuracy |
| --- | --- |
| CAIS-P | 0.767 |
| CBCL-Anxious/Depressed | 0.570 |
| MASC-P | 0.597 |
| PARS-severity mean score | 0.697 |

*Note.* CAIS-P: Child Anxiety Impact Scale, Parent Report; CBCL-Anxious/Depressed subscale; MASC-P: Multidimensional Anxiety Scale for Children, Parent Version; PARS: Pediatric Anxiety Rating Scale severity mean score

4.5. Imputed results for paired comparisons of diagnostic accuracy of predicting recovery from all anxiety diagnoses across measures

| Comparisons | DeLong statistics | p values |
| --- | --- | --- |
| CAIS-P vs CBCL-Anxious/Depressed | Z = 5.124 | p<0.001* |
| CAIS-P vs MASC-P | Z = 3.871 | p<0.001* |
| CAIS-P vs PARS | Z = 1.717 | p=0.086 |
| CBCL-Anxious/Depressed vs MASC-P | Z = -0.630 | p = 0.529 |
| CBCL-Anxious/Depressed vs PARS | Z = -3.354 | p<0.001* |
| MASC-P vs PARS | Z = -2.247 | **p= 0.025 (trend)** |

*p<0.00833, significance level after Bonferroni correction

*Note.* CAIS-P: Child Anxiety Impact Scale, Parent Report; CBCL-Anxious/Depressed subscale; MASC-P: Multidimensional Anxiety Scale for Children, Parent Version; PARS: Pediatric Anxiety Rating Scale severity mean score

4.6. Imputed results for subgroup comparisons of diagnostic accuracy of predicting recovery from all anxiety diagnoses

4.6.1. Comparisons of diagnostic accuracy across different levels of autism traits

| Measures | Different levels of autism traits | Imputed Data  Diagnostic Accuracy |
| --- | --- | --- |
| CAIS-P | Mild | 0.779 |
|  | Moderate | 0.780 |
|  | Severe | 0.798 |
| CBCL-Anxious/Depressed | Mild | 0.616 |
|  | Moderate | 0.569 |
|  | Severe | 0.566 |
| MASC-P | Mild | 0.592 |
|  | Moderate | 0.678 |
|  | Severe | 0.614 |
| PARS-severity mean score | Mild | 0.637 |
|  | Moderate | 0.727 |
|  | Severe | 0.768 |

Mild: SRS scores at or below the bottom 30% of the sample; Moderate: SRS scores between the middle 40% of the sample; severe: SRS scores at or above the top 30% of the sample

*Note.* CAIS-P: Child Anxiety Impact Scale, Parent Report; CBCL-Anxious/Depressed subscale; MASC-P: Multidimensional Anxiety Scale for Children, Parent Version; PARS: Pediatric Anxiety Rating Scale severity mean score

| **DeLong tests on imputed data** | | | |
| --- | --- | --- | --- |
| Measures | sub-group analysis | DeLong statistics | p values |
| CAIS-P | Mild versus moderate | D = -0.013 | p=0.990 |
|  | Mild versus severe | D = -0.205 | p=0.838 |
|  | Moderate versus severe | D = -0.204 | p=0.839 |
| CBCL-Anxious/Depressed | Mild versus moderate | D = 0.453 | p=0.651 |
|  | Mild versus severe | D =0.455 | p=0.650 |
|  | Moderate versus severe | D =0.029 | p = 0.977 |
| MASC-P | Mild versus moderate | D = -0.751 | p=0.454 |
|  | Mild versus severe | D = -0.197 | p=0.844 |
|  | Moderate versus severe | D = 0.582 | p=0.562 |
| PARS-severity mean score | Mild versus moderate | D = -0.818 | p=0.415 |
|  | Mild versus severe | D = -1.268 | p=0.208 |
|  | Moderate versus severe | D = -0.400 | p=0.690 |

Mild: SRS scores at or below the bottom 30% of the sample; Moderate: SRS scores between the middle 40% of the sample; severe: SRS scores at or above the top 30% of the sample

*Note.* CAIS-P: Child Anxiety Impact Scale, Parent Report; CBCL-Anxious/Depressed subscale; MASC-P: Multidimensional Anxiety Scale for Children, Parent Version; PARS: Pediatric Anxiety Rating Scale severity mean score

4.6.2. Comparisons of diagnostic accuracy across different numbers of baseline comorbidities

| Measures | Different numbers of baseline comorbidities grouping | Imputed Data  Diagnostic accuracy |
| --- | --- | --- |
| CAIS-P | <4 | 0.720 |
|  | [4,6) | 0.728 |
|  | >=6 | 0.775 |
| CBCL-Anxious/Depressed | <4 | 0.532 |
|  | [4,6) | 0.590 |
|  | >=6 | 0.554 |
| MASC-P | <4 | 0.581 |
|  | [4,6) | 0.677 |
|  | >=6 | 0.582 |
| PARS-severity mean score | <4 | 0.629 |
|  | [4,6) | 0.728 |
|  | >=6 | 0.672 |

*Note.* CAIS-P: Child Anxiety Impact Scale, Parent Report; CBCL-Anxious/Depressed subscale; MASC-P: Multidimensional Anxiety Scale for Children, Parent Version; PARS: Pediatric Anxiety Rating Scale severity mean score

| **DeLong test on imputed data** | | | |
| --- | --- | --- | --- |
|  | sub-group analysis | DeLong statistics | p values |
| CAIS-P | less than 4 baseline comorbid anxiety Dx versus 4-6 baseline comorbid anxiety Dx | D =-0.058 | p=0.954 |
|  | less than 4 baseline comorbid anxiety Dx versus more than 6 baseline comorbid anxiety Dx | D = -0.439 | p=0.663 |
|  | 4-6 baseline comorbid anxiety Dx versus more than 6 baseline comorbid anxiety Dx | D = -0.545 | p=0.587 |
| CBCL-Anxious/Depressed | less than 4 baseline comorbid anxiety Dx versus 4-6 baseline comorbid anxiety Dx | D = -0.414 | p=0.680 |
|  | less than 4 baseline comorbid anxiety Dx versus more than 6 baseline comorbid anxiety Dx | D = -0.171 | p=0.865 |
|  | 4-6 baseline comorbid anxiety Dx versus more than 6 baseline comorbid anxiety Dx | D = 0.351 | p=0.726 |
| MASC-P | less than 4 baseline comorbid anxiety Dx versus 4-6 baseline comorbid anxiety Dx | D = -0.474 | p=0.638 |
|  | less than 4 baseline comorbid anxiety Dx versus more than 6 baseline comorbid anxiety Dx | D = -0.005 | p=0.996 |
|  | 4-6 baseline comorbid anxiety Dx versus more than 6 baseline comorbid anxiety Dx | D = 0.939 | p=0.345 |
| PARS-severity mean score | less than 4 baseline comorbid anxiety Dx versus 4-6 baseline comorbid anxiety Dx | D = -0.586 | p=0.560 |
|  | less than 4 baseline comorbid anxiety Dx versus more than 6 baseline comorbid anxiety Dx | D =-0.260 | p=0.796 |
|  | 4-6 baseline comorbid anxiety Dx versus more than 6 baseline comorbid anxiety Dx | D = 0.563 | p=0.574 |

*Note.* CAIS-P: Child Anxiety Impact Scale, Parent Report; CBCL-Anxious/Depressed subscale; MASC-P: Multidimensional Anxiety Scale for Children, Parent Version; PARS: Pediatric Anxiety Rating Scale severity mean score; Dx: diagnoses

4.6.3. Comparisons of diagnostic accuracy across different levels of verbal abilities

| Measures | Different levels of vocabulary comprehension | Imputed data  diagnostic accuracy |
| --- | --- | --- |
| CAIS-P | Bottom 30% of vocabulary comprehension | 0.694 |
|  | Middle 40% of vocabulary comprehension | 0.789 |
|  | Top 30% of vocabulary comprehension | 0.823 |
| CBCL-Anxious/Depressed | Bottom 30% of vocabulary comprehension | 0.587 |
|  | Middle 40% of vocabulary comprehension | 0.531 |
|  | Top 30% of vocabulary comprehension | 0.568 |
| MASC-P | Bottom 30% of vocabulary comprehension | 0.647 |
|  | Middle 40% of vocabulary comprehension | 0.565 |
|  | Top 30% of vocabulary comprehension | 0.591 |
| PARS-severity mean score | Bottom 30% of vocabulary comprehension | 0.766 |
|  | Middle 40% of vocabulary comprehension | 0.721 |
|  | Top 30% of vocabulary comprehension | 0.604 |

*Note.* CAIS-P: Child Anxiety Impact Scale, Parent Report; CBCL-Anxious/Depressed subscale; MASC-P: Multidimensional Anxiety Scale for Children, Parent Version; PARS: Pediatric Anxiety Rating Scale severity mean score

| **DeLong tests on imputed data** | | | |
| --- | --- | --- | --- |
| Measures | sub-group analysis | DeLong statistics | p values |
| CAIS-P | Low versus Medium | D = -1.005 | p=0.317 |
|  | Low versus High | D = -1.392 | p=0.167 |
|  | Medium versus High | D = -0.374 | p=0.709 |
| CBCL-Anxious/Depressed | Low versus Medium | D = 0.533 | p=0.595 |
|  | Low versus High | D = 0.170 | p=0.865 |
|  | Medium versus High | D = -0.317 | p=0.752 |
| MASC-P | Low versus Medium | D = 0.648 | p=0.518 |
|  | Low versus High | D = 0.4591 | p=0.647 |
|  | Medium versus High | D = -0.186 | p=0.853 |
| PARS-severity mean score | Low versus Medium | D = 0.421 | p=0.675 |
|  | Low versus High | D = 1.427 | p=0.158 |
|  | Medium versus High | D = 0.927 | p=0.356 |

Low: WISC-vocabulary subscale scores at or below the bottom 30%; Medium: WISC-vocabulary subscale scores between the middle 40%; High: WISC-vocabulary subscale scores at or above the top 30%

*Note.* CAIS-P: Child Anxiety Impact Scale, Parent Report; CBCL-Anxious/Depressed subscale; MASC-P: Multidimensional Anxiety Scale for Children, Parent Version; PARS: Pediatric Anxiety Rating Scale severity mean score

4.6.4. Comparisons of diagnostic accuracy across different sex

| Measures | Sex | Imputed data diagnostic accuracy |
| --- | --- | --- |
| CAIS-P | Male | 0.722 |
|  | Female | 0.917 |
| CBCL-Anxious/Depressed | Male | 0.564 |
|  | Female | 0.592 |
| MASC-P | Male | 0.583 |
|  | Female | 0.652 |
| PARS-severity mean score | Male | 0.684 |
|  | Female | 0.759 |

*Note.* CAIS-P: Child Anxiety Impact Scale, Parent Report; CBCL-Anxious/Depressed subscale; MASC-P: Multidimensional Anxiety Scale for Children, Parent Version; PARS: Pediatric Anxiety Rating Scale severity mean score

| **DeLong tests on imputed data** | | | |
| --- | --- | --- | --- |
| Measures | sub-group analysis | DeLong statistics | p values |
| CAIS-P | Male versus female | D =-2.939 | p=0.004* |
| CBCL-Anxious/Depressed | Male versus female | D =-0.267 | p=0.791 |
| MASC-P | Male versus female | D = -0.538 | p=0.593 |
| PARS-severity mean score | Male versus female | D = -0.754 | p=0.454 |

*p<0.00833, significance level after Bonferroni correction

*Note.* CAIS-P: Child Anxiety Impact Scale, Parent Report; CBCL-Anxious/Depressed subscale; MASC-P: Multidimensional Anxiety Scale for Children, Parent Version; PARS: Pediatric Anxiety Rating Scale severity mean score

4.6.5. Comparisons of diagnostic accuracy for those younger than 10 years old, or 10 years old and above

We chose 10 years old as a cutoff for age because it was the closest year to both the upper and lower bound of 30 percentile and also the median.

| Measures | Age | Imputed Data  Diagnostic Accuracy |
| --- | --- | --- |
| CAIS-P | <10 | 0.823 |
|  | >=10 | 0.723 |
| CBCL-Anxious/Depressed | <10 | 0.620 |
|  | >=10 | 0.473 |
| MASC-P | <10 | 0.646 |
|  | >=10 | 0.455 |
| PARS-severity mean score | <10 | 0.702 |
|  | >=10 | 0.690 |

*Note.* CAIS-P: Child Anxiety Impact Scale, Parent Report; CBCL-Anxious/Depressed subscale; MASC-P: Multidimensional Anxiety Scale for Children, Parent Version; PARS: Pediatric Anxiety Rating Scale severity mean score

| **DeLong test on imputed data** | | | |
| --- | --- | --- | --- |
| Measures | sub-group analysis | DeLong statistics | p values |
| CAIS-P | <10 years versus >=10 years | D = 1.397 | p=0.164 |
| CBCL-Anxious/Depressed | <10 years versus >=10 years | D = 1.693 | p=0.092 |
| MASC-P | <10 years versus >=10 years | D = 2.051 | **p=0.042 (trend)** |
| PARS-severity mean score | <10 years versus >=10 years | D =0.132 | p=0.896 |

*Note.* CAIS-P: Child Anxiety Impact Scale, Parent Report; CBCL-Anxious/Depressed subscale; MASC-P: Multidimensional Anxiety Scale for Children, Parent Version; PARS: Pediatric Anxiety Rating Scale severity mean score

4.7. Correlational Analyses for Validities of the Subscales of CAIS-P with imputation

4.7.1. Construct validities of CAIS-P social and school impact scales, when compared to CBCL social competence mean score

|  | Pre-treatment CBCL social competence mean score** | | Post-treatment CBCL social competence mean score** | |
| --- | --- | --- | --- | --- |
|  | Spearman's rho | p value | Spearman's rho | p value |
| Pre-treatment CAIS social impact mean score | -0.146 | **p=0.033 (trend)** | **-0.201** | **p=0.0032***  **- but not relevant** |
| Post-treatment CAIS social impact mean score | -0.030 | p=0.667 | -0.149 | **p=0.030 (trend)** |
| Pre-treatment CAIS school impact mean score | -0.136 | **p=0.047 (trend)** | -0.058 | p=0.398 |
| Post-treatment CAIS school impact mean score | **-0.177** | **p=0.01**  **(trend, but not relevant)** | -0.180 | **p=0.009 (trend)** |

*p<0.00833, significance level after Bonferroni correction

** We chose to use mean rather than total score for the CBCL-social competence subscale as one of the items asked about sibling relationship and we do not want to penalise participants who might be the only child of the family

*Note.* CAIS-P: Child Anxiety Impact Scale, Parent Report; CBCL-Anxious/Depressed subscale; MASC-P: Multidimensional Anxiety Scale for Children, Parent Version; PARS: Pediatric Anxiety Rating Scale severity mean score

4.7.2. Convergent validities across scales with imputation

|  | Pre-treatment CAIS-P | Post-treatment CAIS-P | | Pre-treatment CBCL anxious/depressed -P | | Post-treatment CBCL anxious/depressed -P | |  |
| --- | --- | --- | --- | --- | --- | --- | --- | --- |
|  | Spearman's rho | p value | Spearman's rho | p value | Spearman's rho | p value | Spearman's rho | p value |
| Pre-treatment CAIS-P | / | / | 0.5927 | p<0.001* | 0.423 | p<0.001* | 0.345 | p<0.001* |
| Post-treatment CAIS-P | 0.593 | p<0.001* | / | / | 0.212 | p=0.002* | 0.555 | p<0.001* |
| Pre-treatment CBCL anxious/depressed -P | 0.423 | p<0.001* | 0.212 | p=0.002* | / | p<0.001* | 0.587 | p<0.001* |
| Post-treatment CBCL anxious/depressed -P | 0.345 | p<0.001* | 0.555 | p<0.001* | 0.587 | p<0.001* | / | / |
| Pre-treatment MASC-P | 0.439 | p<0.001* | 0.297 | p<0.001* | 0.514 | p<0.001* | 0.467 | p<0.001* |
| Post-treatment MASC-P | 0.351 | p<0.001* | 0.497 | p<0.001* | 0.368 | p<0.001* | 0.631 | p<0.001* |
| Pre-treatment PARS severity mean scores | 0.366305 | p<0.001* | 0.325 | p<0.001* | 0.242 | p<0.001* | 0.328 | p<0.001* |
| Post-treatment PARS severity mean scores | 0.438 | p<0.001* | 0.641 | p<0.001* | 0.308 | p<0.001* | 0.573 | p<0.001* |

*p<0.00833, significance level after Bonferroni correction

*Note.* CAIS-P: Child Anxiety Impact Scale, Parent Report; CBCL-Anxious/Depressed subscale; MASC-P: Multidimensional Anxiety Scale for Children, Parent Version; PARS: Pediatric Anxiety Rating Scale severity mean score

Appendix 5. Correlational Analyses for Construct Validity of the Subscales of the CAIS-P in complete case analysis

|  | Pre-treatment CBCL social competence mean score** | | Post-treatment CBCL social competence mean score** | |
| --- | --- | --- | --- | --- |
|  | Spearman's rho | p value | Spearman's rho | p value |
| Pre-treatment CAIS social impact mean score | -0.138 | p=0.047 | -0.086 | p=0.323 |
| Post-treatment CAIS social impact mean score | 0.544 | p<0.001*  (but not relevant) | -0.079 | p=0.363 |
| Pre-treatment CAIS school impact mean score | -0.149 | p=0.032 | -0.0803 | p=0.358 |
| Post-treatment CAIS school impact mean score | -0.156 | p=0.074 | -0.180 | p=0.037 |

*p<0.00833, significance level after Bonferroni correction

** We chose to use mean rather than total score for the CBCL-social competence subscale as one of the items asked about sibling relationship and we do not want to penalise participants who might be the only child of the family

*Note.* CAIS-P: Child Anxiety Impact Scale, Parent Report; CBCL-Anxious/Depressed subscale; MASC-P: Multidimensional Anxiety Scale for Children, Parent Version; PARS: Pediatric Anxiety Rating Scale severity mean score

Appendix 6. 3-Factor Confirmation Factor Analysis of CAIS-P in complete case analysis

6.1. Pre-treatment treatment CAIS-P Factor Loadings to the Original 3-Factor Model

$\chi^{2}=$1129.79, df=345, p<0.0005

CMIN: 1129.79, p<0.0005

CFI=0.526

RMSEA:=0.108, p close<0.0005


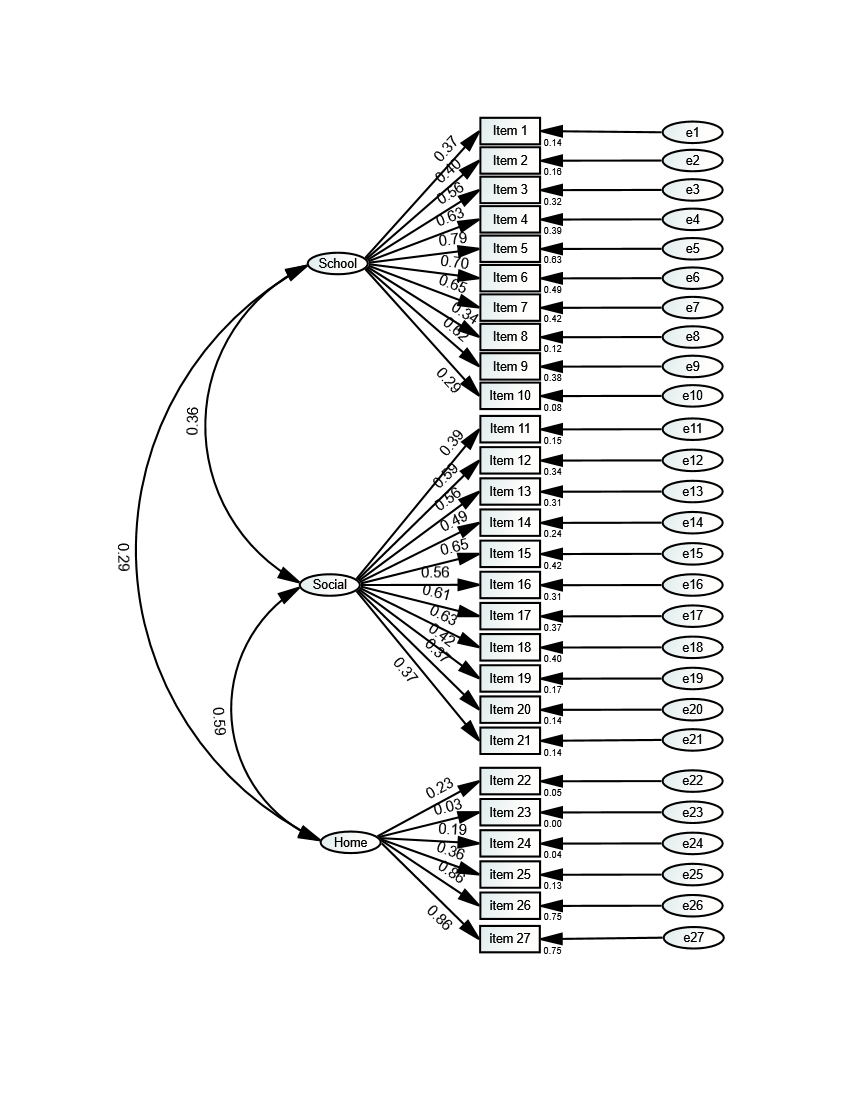


6.2. Post-treatment CAIS-P Factor Loadings to the Original 3-Factor Model

$\chi^{2}=$917.25, Df=321, p<0.0005

CMIN=917.25, Df=321, p<0.0005

CFI=0.56=0.566

RMSEA=0.120, pclose<0.0005


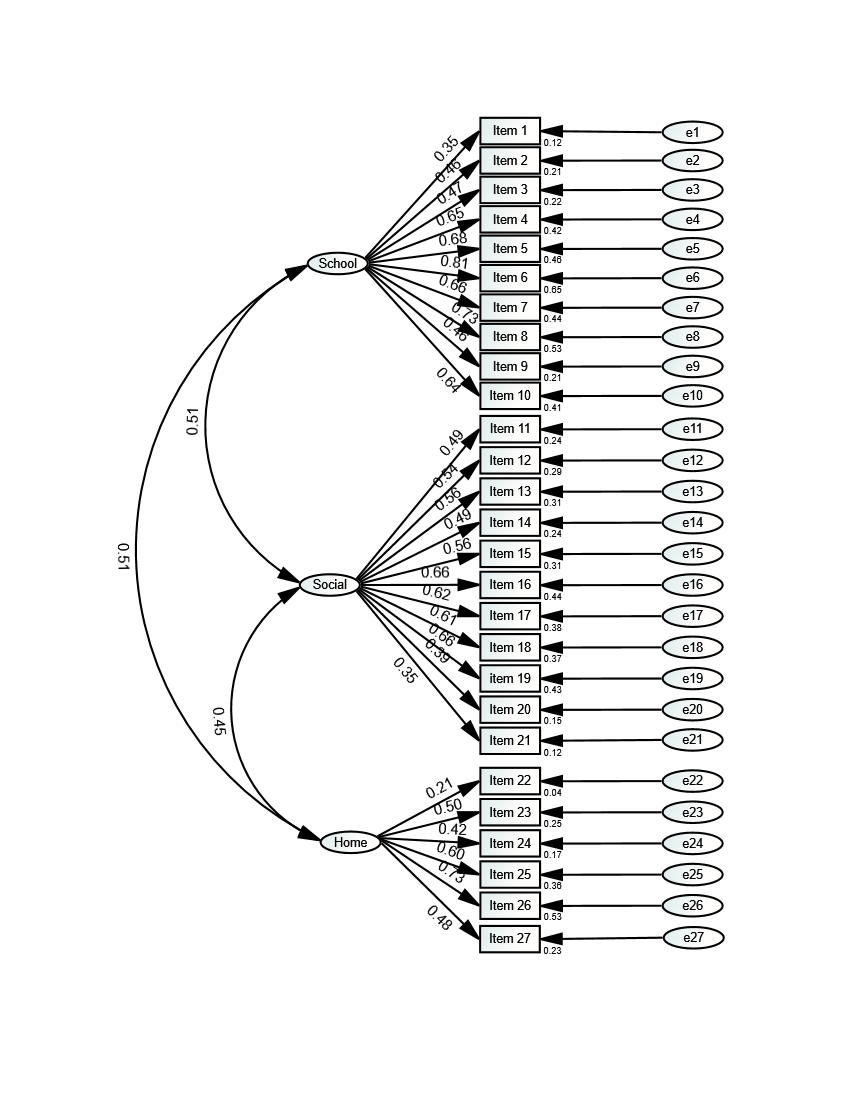


Appendix 7. Exploratory Factor Analysis of CAIS-P in complete case analysis

7.1. EFA of Pre-treatment CAIS-P Scores

| Component* | Initial Eigenvalues | | |
| --- | --- | --- | --- |
|  | Total | % of Variance | Cumulative % |
| 1 | 5.904 | 21.865 | 21.865 |
| 2 | 2.809 | 10.403 | 32.268 |
| 3 | 2.060 | 7.628 | 39.896 |
| 4 | 1.772 | 6.563 | 46.459 |
| 5 | 1.407 | 5.210 | 51.669 |
| 6 | 1.345 | 4.980 | 56.649 |
| 7 | 1.189 | 4.404 | 61.053 |
| 8 | 1.019 | 3.774 | 64.826 |

*All components with Eigenvalues >=1

| **Component Matrix^a^** | | | | | | | | |
| --- | --- | --- | --- | --- | --- | --- | --- | --- |
|  | Component | | | | | | | |
|  | 1 | 2 | 3 | 4 | 5 | 6 | 7 | 8 |
| CAIS-P 1. Getting to school on time in the morning | .455 | .172 | .341 | -.054 | -.123 | -.049 | -.120 | -.005 |
| CAIS-P 2. Giving oral reports or reading out loud | .516 | .048 | -.360 | -.231 | -.134 | .086 | .371 | -.030 |
| CAIS-P 3. Writing in class | .349 | .522 | -.229 | -.028 | .183 | -.210 | -.048 | -.003 |
| CAIS-P 4. Taking tests or exams | .470 | .465 | -.250 | .061 | -.029 | -.087 | .104 | .266 |
| CAIS-P 5. Completing assignments in class | .544 | .609 | -.103 | .023 | .080 | -.067 | -.024 | .078 |
| CAIS-P 6. Doing homework | .492 | .604 | .163 | .117 | -.068 | .022 | -.081 | -.100 |
| CAIS-P 7. Getting good grades | .449 | .522 | -.215 | .046 | .074 | -.161 | .172 | .048 |
| CAIS-P 8. Doing fun things during recess or free time | .466 | -.013 | -.299 | .091 | -.048 | .352 | -.456 | .134 |
| CAIS-P 9. Concentrating on his/her work | .454 | .511 | .058 | .129 | .045 | .062 | .005 | -.358 |
| CAIS-P 10. Eating lunch with other kids | .443 | -.065 | -.284 | -.046 | -.025 | .573 | -.370 | -.008 |
| CAIS-P 11. Making new friends | .431 | -.224 | -.362 | -.187 | -.114 | .408 | .135 | .170 |
| CAIS-P 12. Leaving the house | .585 | -.190 | .294 | .036 | -.212 | .058 | -.223 | -.346 |
| CAIS-P 13. Talking on the phone | .534 | -.320 | -.180 | -.230 | .010 | .065 | .265 | -.254 |
| CAIS-P 14. Being with a group of strangers | .470 | -.312 | -.300 | -.136 | -.175 | .076 | .436 | -.148 |
| CAIS-P 15. Going to a friend's house during the day | .516 | -.437 | .013 | .282 | -.166 | -.225 | -.094 | .145 |
| CAIS-P 16. Spending the night at a friend's house | .459 | -.365 | .090 | .288 | -.335 | -.216 | -.056 | .182 |
| CAIS-P 17. Going to a sports event or ball game | .535 | -.290 | .015 | .023 | -.168 | -.340 | -.139 | -.199 |
| CAIS-P 18. Going shopping or trying on clothes | .661 | -.031 | .146 | .008 | -.163 | -.159 | -.004 | -.326 |
| CAIS-P 19. Going on a date | .355 | -.313 | -.061 | .616 | .457 | .066 | .131 | -.113 |
| CAIS-P 20. Having a boyfriend/girlfriend | .351 | -.229 | .032 | .598 | .543 | .086 | .141 | -.002 |
| CAIS-P 21. Eating in public | .430 | -.089 | .025 | .008 | .242 | .100 | -.179 | .164 |
| CAIS-P 22. Getting ready for bed at night | .440 | .123 | .512 | .068 | -.232 | .147 | .085 | .296 |
| CAIS-P 23. Sleeping at night | .238 | .076 | .485 | .282 | -.265 | .226 | .397 | .313 |
| CAIS-P 24. Getting along wth his/her brothers or sisters | .128 | -.024 | .498 | -.363 | .382 | .203 | .171 | -.067 |
| CAIS-P 25. Getting along with his/her parents | .379 | -.023 | .558 | -.389 | .224 | .214 | -.040 | -.047 |
| CAIS-P 26. Visiting relatives | .597 | -.291 | -.073 | -.337 | .220 | -.347 | -.127 | .226 |
| CAIS-P 27. Having relatives visit | .542 | -.234 | .065 | -.423 | .321 | -.275 | -.044 | .255 |
| Extraction Method: Principal Component Analysis. | | | | | | | | |
| a. 8 components extracted. | | | | | | | | |

6.2. EFA of Post-treatment CAIS-P Scores

| Component* | Initial Eigenvalues | | |
| --- | --- | --- | --- |
|  | Total | % of Variance | Cumulative % |
| 1 | 6.929 | 25.663 | 25.663 |
| 2 | 2.531 | 9.373 | 35.036 |
| 3 | 2.336 | 8.650 | 43.686 |
| 4 | 1.797 | 6.655 | 50.342 |
| 5 | 1.464 | 5.421 | 55.763 |
| 6 | 1.341 | 4.965 | 60.728 |
| 7 | 1.217 | 4.506 | 65.234 |
| 8 | 1.002 | 3.713 | 68.947 |

*All components with Eigenvalues >=1

| **Component Matrix^a^** | | | | | | | | |
| --- | --- | --- | --- | --- | --- | --- | --- | --- |
|  | Component | | | | | | | |
|  | 1 | 2 | 3 | 4 | 5 | 6 | 7 | 8 |
| CAIS-P 1. Getting to school on time in the morning | 0.553 | -0.107 | 0.386 | 0.035 | -0.291 | 0.009 | -0.114 | 0.015 |
| CAIS-P 2. Giving oral reports or reading out loud | 0.539 | -0.041 | -0.263 | -0.137 | 0.283 | 0.049 | -0.241 | -0.035 |
| CAIS-P 3. Writing in class | 0.505 | -0.431 | -0.297 | -0.167 | 0.069 | 0.22 | -0.146 | 0.049 |
| CAIS-P 4. Taking tests or exams | 0.545 | -0.463 | -0.266 | -0.019 | 0.039 | 0.045 | -0.226 | 0.105 |
| CAIS-P 5. Completing assignments in class | 0.63 | -0.53 | -0.143 | -0.047 | -0.012 | 0.017 | -0.077 | 0.116 |
| CAIS-P 6. Doing homework | 0.532 | -0.496 | 0.15 | -0.033 | -0.006 | -0.032 | 0.133 | 0.121 |
| CAIS-P 7. Getting good grades | 0.599 | -0.494 | -0.105 | -0.111 | 0.035 | 0.213 | 0.101 | 0.011 |
| CAIS-P 8. Doing fun things during recess or free time | 0.567 | -0.034 | -0.197 | -0.067 | -0.351 | -0.356 | 0.037 | -0.361 |
| CAIS-P 9. Concentrating on his/her work | 0.553 | -0.458 | 0.065 | 0.006 | -0.008 | -0.061 | 0.193 | 0.036 |
| CAIS-P 10. Eating lunch with other kids | 0.535 | 0.146 | 0.124 | -0.241 | -0.52 | -0.185 | -0.101 | -0.024 |
| CAIS-P 11. Making new friends | 0.506 | 0.122 | -0.389 | -0.153 | 0.016 | -0.104 | 0.176 | -0.072 |
| CAIS-P 12. Leaving the house | 0.586 | 0.294 | 0.327 | -0.084 | -0.175 | 0.07 | 0.004 | 0.04 |
| CAIS-P 13. Talking on the phone | 0.421 | 0.354 | 0.088 | -0.12 | 0.224 | -0.15 | 0.341 | 0.533 |
| CAIS-P 14. Being with a group of strangers | 0.485 | 0.373 | -0.233 | -0.067 | 0.318 | -0.014 | 0.068 | 0.413 |
| CAIS-P 15. Going to a friend's house during the day | 0.543 | 0.324 | -0.423 | -0.08 | -0.049 | -0.033 | 0.327 | -0.2 |
| CAIS-P 16. Spending the night at a friend's house | 0.522 | 0.29 | -0.345 | -0.054 | 0.085 | 0.226 | 0.32 | -0.3 |
| CAIS-P 17. Going to a sports event or ball game | 0.523 | 0.462 | 0.05 | -0.272 | -0.144 | 0.044 | -0.006 | 0.015 |
| CAIS-P 18. Going shopping or trying on clothes | 0.642 | 0.255 | 0.004 | -0.009 | -0.13 | 0.121 | -0.041 | 0.069 |
| CAIS-P 19. Going on a date | 0.336 | 0.246 | -0.362 | 0.753 | -0.192 | 0.082 | -0.159 | 0.064 |
| CAIS-P 20. Having a boyfriend/girlfriend | 0.331 | 0.134 | -0.347 | 0.773 | -0.166 | 0.098 | -0.163 | 0.141 |
| CAIS-P 21. Eating in public | 0.338 | 0.254 | 0.311 | -0.252 | -0.279 | -0.002 | -0.401 | 0.211 |
| CAIS-P 22. Getting ready for bed at night | 0.396 | 0.001 | 0.512 | 0.158 | -0.09 | 0.478 | 0.165 | -0.049 |
| CAIS-P 23. Sleeping at night | 0.305 | 0.107 | 0.459 | 0.134 | 0.229 | 0.566 | 0.122 | -0.188 |
| CAIS-P 24. Getting along wth his/her brothers or sisters | 0.365 | -0.143 | 0.33 | 0.36 | 0.213 | -0.529 | 0.185 | -0.008 |
| CAIS-P 25. Getting along with his/her parents | 0.464 | -0.162 | 0.463 | 0.37 | -0.001 | -0.267 | 0.264 | -0.041 |
| CAIS-P 26. Visiting relatives | 0.565 | 0.19 | 0.168 | 0.023 | 0.469 | -0.194 | -0.278 | -0.325 |
| CAIS-P 27. Having relatives visit | 0.554 | 0.212 | 0.229 | 0.024 | 0.448 | -0.124 | -0.439 | -0.132 |
| Extraction Method: Principal Component Analysis. | | | | | | | | |
| a. 8 components extracted. | | | | | | | | |

Appendix 8. DeLong Test of Complete Case Subgroup Analyses of Diagnostic Accuracy in complete case analysis

8.1 Complete case analysis of paired comparisons of diagnostic accuracy of predicting recovery from primary diagnoses across measures

| Comparisons | DeLong statistics | p values |
| --- | --- | --- |
| CAIS-P vs CBCL-Anxious/Depressed | Z = -0.812 | p = 0.417 |
| CAIS-P vs MASC-P | Z = -0.430 | p = 0.667 |
| CAIS-P vs PARS | Z = 0.418 | p= 0.676 |
| CBCL-Anxious/Depressed vs MASC-P | Z = 0.595 | p= 0.552 |
| CBCL-Anxious/Depressed vs PARS | Z = 1.674 | **p = 0.094 (trend)** |
| MASC-P vs PARS | Z = 1.386 | p= 0.166 |

*Note.* CAIS-P: Child Anxiety Impact Scale, Parent Report; CBCL-Anxious/Depressed subscale; MASC-P: Multidimensional Anxiety Scale for Children, Parent Version; PARS: Pediatric Anxiety Rating Scale severity mean score

8.2. Complete case analysis for subgroup comparisons of diagnostic accuracy of predicting recovery from primary anxiety diagnoses

8.2.1. Comparisons among different measures

|  | Recovery from primary anxiety diagnoses | | Recovery from all anxiety diagnoses | |
| --- | --- | --- | --- | --- |
|  | DeLong statistics | Significance levels | DeLong statistics | Significance levels |
| CAIS-P vs CBCL-Anxious/Depressed | Z = -0.812 | p = 0.417 | Z = 1.460 | p = 0.145 |
| CAIS-P vs MASC-P | Z = -0.430 | p = 0.667 | Z = 1.344 | p = 0.179 |
| CAIS-P vs PARS | Z = 0.4183 | p= 0.676 | Z = 1.298 | p = 0.194 |
| CBCL-Anxious/Depressed vs MASC-P | Z = 0.595 | p= 0.552 | Z = -0.037 | p = 0.971 |
| CBCL-Anxious/Depressed vs PARS | Z = 1.674 | **p = 0.094 (trend)** | Z = 0.288 | p = 0.773 |
| MASC-P vs PARS | Z = 1.386 | p= 0.166 | Z = 0.142 | p = 0.887 |

*Note.* CAIS-P: Child Anxiety Impact Scale, Parent Report; CBCL-Anxious/Depressed subscale; MASC-P: Multidimensional Anxiety Scale for Children, Parent Version; PARS: Pediatric Anxiety Rating Scale severity mean score

8.2.2. Comparisons of diagnostic accuracy across different levels of autism traits

| Measures | Different levels of autism traits | Complete Data  Diagnostic Accuracy |
| --- | --- | --- |
| CAIS-P | Mild | 0.730 |
|  | Moderate | 0.521 |
|  | Severe | 0.586 |
| CBCL-Anxious/Depressed | Mild | 0.601 |
|  | Moderate | 0.711 |
|  | Severe | 0.59 |
| MASC-P | Mild | 0.618 |
|  | Moderate | 0.482 |
|  | Severe | 0.677 |
| PARS-severity mean score | Mild | 0.671 |
|  | Moderate | 0.561 |
|  | Severe | 0.533 |

Mild: SRS scores at or below the bottom 30% of the sample; Moderate: SRS scores between the middle 40% of the sample; severe: SRS scores at or above the top 30% of the sample

*Note.* CAIS-P: Child Anxiety Impact Scale, Parent Report; CBCL-Anxious/Depressed subscale; MASC-P: Multidimensional Anxiety Scale for Children, Parent Version; PARS: Pediatric Anxiety Rating Scale severity mean score

| **DeLong tests on complete data** | | | |
| --- | --- | --- | --- |
| Measures | sub-group analysis | DeLong statistics | p values |
| CAIS-P | Mild versus moderate | Z = 1.103 | P= 0.273 |
|  | Mild versus severe | Z = 0.690 | p = 0.493 |
|  | Moderate versus severe | Z = -0.264 | p = 0.792 |
| CBCL-Anxious/Depressed | Mild versus moderate | Z = -0.516 | p = 0.608 |
|  | Mild versus severe | Z = 0.046 | p = 0.963 |
|  | Moderate versus severe | Z = 0.646 | p = 0.520 |
| MASC-P | Mild versus moderate | Z = 0.723 | p= 0.472 |
|  | Mild versus severe | Z = -0.326 | p = 0.745 |
|  | Moderate versus severe | Z = -1.010 | p = 0.316 |
| PARS-severity mean score | Mild versus moderate | Z = 0.540 | p = 0.591 |
|  | Mild versus severe | Z = 0.657 | p = 0.513 |
|  | Moderate versus severe | Z = 0.141 | p = 0.889 |

Mild: SRS scores at or below the bottom 30% of the sample; Moderate: SRS scores between the middle 40% of the sample; severe: SRS scores at or above the top 30% of the sample

*Note.* CAIS-P: Child Anxiety Impact Scale, Parent Report; CBCL-Anxious/Depressed subscale; MASC-P: Multidimensional Anxiety Scale for Children, Parent Version; PARS: Pediatric Anxiety Rating Scale severity mean score

8.2.3. Comparisons of diagnostic accuracy across different numbers of baseline comorbidities

| Measures | Different numbers of baseline comorbidities grouping | Complete Data  Diagnostic accuracy |
| --- | --- | --- |
| CAIS-P | <4 | 0.634 |
|  | [4,6) | 0.651 |
|  | >=6 | 0.677 |
| CBCL-Anxious/Depressed | <4 | 0.716 |
|  | [4,6) | 0.634 |
|  | >=6 | 0.59 |
| MASC-P | <4 | 0.857 |
|  | [4,6) | 0.591 |
|  | >=6 | 0.501 |
| PARS-severity mean score | <4 | 0.542 |
|  | [4,6) | 0.531 |
|  | >=6 | 0.603 |

*Note.* CAIS-P: Child Anxiety Impact Scale, Parent Report; CBCL-Anxious/Depressed subscale; MASC-P: Multidimensional Anxiety Scale for Children, Parent Version; PARS: Pediatric Anxiety Rating Scale severity mean score

| **DeLong test on complete data** | | | |
| --- | --- | --- | --- |
|  | sub-group analysis | DeLong statistics | p values |
| CAIS-P | less than 4 baseline comorbid anxiety Dx versus 4-6 baseline comorbid anxiety Dx | Z = -0.0522 | p = 0.959 |
|  | less than 4 baseline comorbid anxiety Dx versus more than 6 baseline comorbid anxiety Dx | Z = -0.145 | p = 0.886 |
|  | 4-6 baseline comorbid anxiety Dx versus more than 6 baseline comorbid anxiety Dx | Z = -0.147 | p = 0.883 |
| CBCL-Anxious/Depressed | less than 4 baseline comorbid anxiety Dx versus 4-6 baseline comorbid anxiety Dx | Z = 0.418 | p = 0.677 |
|  | less than 4 baseline comorbid anxiety Dx versus more than 6 baseline comorbid anxiety Dx | Z = 0.698 | p = 0.487 |
|  | 4-6 baseline comorbid anxiety Dx versus more than 6 baseline comorbid anxiety Dx | Z = 0.236 | p = 0.814 |
| MASC-P | less than 4 baseline comorbid anxiety Dx versus 4-6 baseline comorbid anxiety Dx | Z = 1.584 | p = 0.119 |
|  | less than 4 baseline comorbid anxiety Dx versus more than 6 baseline comorbid anxiety Dx | Z = 2.886 | p = 0.005* |
|  | 4-6 baseline comorbid anxiety Dx versus more than 6 baseline comorbid anxiety Dx | Z = 0.486 | p = 0.628 |
| PARS-severity mean score | less than 4 baseline comorbid anxiety Dx versus 4-6 baseline comorbid anxiety Dx | Z = 0.056 | p = 0.955 |
|  | less than 4 baseline comorbid anxiety Dx versus more than 6 baseline comorbid anxiety Dx | Z = -0.403 | p = 0.688 |
|  | 4-6 baseline comorbid anxiety Dx versus more than 6 baseline comorbid anxiety Dx | Z = -0.408 | p = 0.684 |

*Note.* CAIS-P: Child Anxiety Impact Scale, Parent Report; CBCL-Anxious/Depressed subscale; MASC-P: Multidimensional Anxiety Scale for Children, Parent Version; PARS: Pediatric Anxiety Rating Scale severity mean score; Dx: diagnoses

8.2.4. Comparisons of diagnostic accuracy across different levels of verbal abilities

| Measures | Different levels of vocabulary comprehension | Complete Data  Diagnostic Accuracy |
| --- | --- | --- |
| CAIS-P | Below sample average | 0.503 |
|  | At or above sample average | 0.631 |
| CBCL-Anxious/Depressed | Below sample average | 0.560 |
|  | At or above sample average | 0.652 |
| MASC-P | Below sample average | 0.588 |
|  | At or above sample average | 0.558 |
| PARS-severity mean score | Below sample average | 0.471 |
|  | At or above sample average | 0.469 |

*Note.* CAIS-P: Child Anxiety Impact Scale, Parent Report; CBCL-Anxious/Depressed subscale; MASC-P: Multidimensional Anxiety Scale for Children, Parent Version; PARS: Pediatric Anxiety Rating Scale severity mean score

| **DeLong tests on complete data** | | | |
| --- | --- | --- | --- |
| Measures | sub-group analysis | DeLong statistics | p values |
| CAIS-P | Below Sample Average versus Sample average or Above average | Z = -0.643 | p = 0.521 |
| CBCL-Anxious/Depressed | Below Sample Average versus Sample average or Above average | Z = -0.527 | p = 0.599 |
| MASC-P | Below Sample Average versus Sample average or Above average | Z = 0.167 | p = 0.867 |
|  | Below Sample Average versus Sample average or Above average | Z = 0.010 | p = 0.992 |
| PARS-severity mean score | Below Sample Average versus Sample average or Above average | Z = -0.643 | p = 0.521 |

Low: WISC-vocabulary subscale scores at or below the bottom 30%; Medium: WISC-vocabulary subscale scores between the middle 40%; High: WISC-vocabulary subscale scores at or above the top 30%

*p<0.00833, significance level after Bonferroni correction

*Note.* CAIS-P: Child Anxiety Impact Scale, Parent Report; CBCL-Anxious/Depressed subscale; MASC-P: Multidimensional Anxiety Scale for Children, Parent Version; PARS: Pediatric Anxiety Rating Scale severity mean score

8.2.5 Comparisons of diagnostic accuracy across different sex

| Measures | Sex | Complete Data Diagnostic Accuracy |
| --- | --- | --- |
| CAIS-P | Male | 0.481 |
|  | Female | 0.622 |
| CBCL-Anxious/Depressed | Male | 0.628 |
|  | Female | 0.609 |
| MASC-P | Male | 0.614 |
|  | Female | 0.524 |
| PARS-severity mean score | Male | 0.535 |
|  | Female | 0.722 |

*Note.* CAIS-P: Child Anxiety Impact Scale, Parent Report; CBCL-Anxious/Depressed subscale; MASC-P: Multidimensional Anxiety Scale for Children, Parent Version; PARS: Pediatric Anxiety Rating Scale severity mean score

| **DeLong tests on complete data** | | | |
| --- | --- | --- | --- |
| Measures | sub-group analysis | DeLong statistics | p values |
| CAIS-P | Male versus female | Z = -0.469 | p = 0.642 |
| CBCL-Anxious/Depressed | Male versus female | Z = 0.095 | p = 0.924 |
| MASC-P | Male versus female | Z = 0.401 | p = 0.691 |
| PARS-severity mean score | Male versus female | Z = -1.193 | p = 0.238 |

*Note.* CAIS-P: Child Anxiety Impact Scale, Parent Report; CBCL-Anxious/Depressed subscale; MASC-P: Multidimensional Anxiety Scale for Children, Parent Version; PARS: Pediatric Anxiety Rating Scale severity mean score

8.2.6. Comparisons of diagnostic accuracy for those younger than 10 years old, or 10 years old and above

We chose 10 years old as a cutoff for age because it was the closest year to both the upper and lower bound of 30 percentile and also the median.

| Measures | Age | Complete Data  Diagnostic Accuracy |
| --- | --- | --- |
| CAIS-P | <10 | 0.505 |
|  | >=10 | 0.675 |
| CBCL-Anxious/Depressed | <10 | 0.568 |
|  | >=10 | 0.702 |
| MASC-P | <10 | 0.620 |
|  | >=10 | 0.534 |
| PARS-severity mean score | <10 | 0.489 |
|  | >=10 | 0.641 |

*Note.* CAIS-P: Child Anxiety Impact Scale, Parent Report; CBCL-Anxious/Depressed subscale; MASC-P: Multidimensional Anxiety Scale for Children, Parent Version; PARS: Pediatric Anxiety Rating Scale severity mean score

| **DeLong test on complete data** | | | |
| --- | --- | --- | --- |
| Measures | sub-group analysis | DeLong statistics | p values |
| CAIS-P | <10 years versus >=10 years | Z = -1.081 | p = 0.282 |
| CBCL-Anxious/Depressed | <10 years versus >=10 years | Z = 0.797 | p = 0.427 |
| MASC-P | <10 years versus >=10 years | Z = 0.512 | p = 0.610 |
| PARS-severity mean score | <10 years versus >=10 years | Z = -0.890 | p = 0.375 |

*Note.* CAIS-P: Child Anxiety Impact Scale, Parent Report; CBCL-Anxious/Depressed subscale; MASC-P: Multidimensional Anxiety Scale for Children, Parent Version; PARS: Pediatric Anxiety Rating Scale severity mean score

8.3. Complete case analysis for paired comparisons of diagnostic accuracy of predicting recovery from all anxiety diagnoses across measures

| Comparisons | DeLong statistics | p values |
| --- | --- | --- |
| CAIS-P vs CBCL-Anxious/Depressed | Z = 1.459 | p = 0.145 |
| CAIS-P vs MASC-P | Z = 1.344 | p = 0.179 |
| CAIS-P vs PARS | Z = 1.298 | p = 0.194 |
| CBCL-Anxious/Depressed vs MASC-P | Z = -0.037 | p = 0.971 |
| CBCL-Anxious/Depressed vs PARS | Z = 0.288 | p = 0.773 |
| MASC-P vs PARS | Z = 0.142 | p = 0.887 |

*Note.* CAIS-P: Child Anxiety Impact Scale, Parent Report; CBCL-Anxious/Depressed subscale; MASC-P: Multidimensional Anxiety Scale for Children, Parent Version; PARS: Pediatric Anxiety Rating Scale severity mean score

8.4. Complete case analysis for subgroup comparisons of diagnostic accuracy of predicting recovery from all anxiety diagnoses

8.4.1. Complete case analysis of diagnostic accuracy across different levels of autism traits

| Measures | Different levels of autism traits | Complete Data  Diagnostic Accuracy |
| --- | --- | --- |
| CAIS-P | Mild | 0.765 |
|  | Moderate | 0.800 |
|  | Severe | 0.969 |
| CBCL-Anxious/Depressed | Mild | 0.558 |
|  | Moderate | 0.527 |
|  | Severe | 0.544 |
| MASC-P | Mild | 0.425 |
|  | Moderate | 0.707 |
|  | Severe | 0.721 |
| PARS-severity mean score | Mild | 0.634 |
|  | Moderate | 0.422 |
|  | Severe | 0.891 |

Mild: SRS scores at or below the bottom 30% of the sample; Moderate: SRS scores between the middle 40% of the sample; severe: SRS scores at or above the top 30% of the sample

*Note.* CAIS-P: Child Anxiety Impact Scale, Parent Report; CBCL-Anxious/Depressed subscale; MASC-P: Multidimensional Anxiety Scale for Children, Parent Version; PARS: Pediatric Anxiety Rating Scale severity mean score

| **DeLong tests on complete data** | | | |
| --- | --- | --- | --- |
| Measures | sub-group analysis | DeLong statistics | p values |
| CAIS-P | Mild versus moderate | Z = -0.201 | p = 0.841 |
|  | Mild versus severe | N/A (insufficient data; insufficient heterogeneity in ROCs) | N/A |
|  | Moderate versus severe | N/A | N/A |
| CBCL-Anxious/Depressed | Mild versus moderate | Z = 0.154 | p = 0.878 |
|  | Mild versus severe | N/A (insufficient data; insufficient heterogeneity in ROCs) | N/A |
|  | Moderate versus severe | N/A | N/A |
| MASC-P | Mild versus moderate | Z = -1.068 | p-value = 0.288 |
|  | Mild versus severe | N/A (insufficient data; insufficient heterogeneity in ROCs) | N/A |
|  | Moderate versus severe | N/A | N/A |
| PARS-severity mean score | Mild versus moderate | N/A (insufficient data; insufficient heterogeneity in ROCs) | N/A |
|  | Mild versus severe | N/A | N/A |
|  | Moderate versus severe | Z = -2.261 | **p = 0.027 (trend)** |

Mild: SRS scores at or below the bottom 30% of the sample; Moderate: SRS scores between the middle 40% of the sample; severe: SRS scores at or above the top 30% of the sample

*Note.* CAIS-P: Child Anxiety Impact Scale, Parent Report; CBCL-Anxious/Depressed subscale; MASC-P: Multidimensional Anxiety Scale for Children, Parent Version; PARS: Pediatric Anxiety Rating Scale severity mean score

8.4.2. Comparisons of diagnostic accuracy across different numbers of baseline comorbidities

| Measures | Different numbers of baseline comorbidities grouping | Complete Data  Diagnostic accuracy |
| --- | --- | --- |
| CAIS-P | <4 | 0.655 |
|  | [4,6) | N/A |
|  | >=6 | 0.770 |
| CBCL-Anxious/Depressed | <4 | 0.583 |
|  | [4,6) | 0.744 |
|  | >=6 | 0.475 |
| MASC-P | <4 | 0.862 |
|  | [4,6) | 0.947 |
|  | >=6 | 0.455 |
| PARS-severity mean score | <4 | 0.629 |
|  | [4,6) | 0.949 |
|  | >=6 | 0.394 |

*No DeLong test was conducted due to insufficient data

*Note.* CAIS-P: Child Anxiety Impact Scale, Parent Report; CBCL-Anxious/Depressed subscale; MASC-P: Multidimensional Anxiety Scale for Children, Parent Version; PARS: Pediatric Anxiety Rating Scale severity mean score

8.4.3. Comparisons of diagnostic accuracy across different levels of verbal abilities

| Measures | Different levels of vocabulary comprehension | Complete data  diagnostic accuracy |
| --- | --- | --- |
| CAIS-P | Belove Sample Average | 0.664 |
|  | At Sample average or Above average | 0.861 |
| CBCL-Anxious/Depressed | Belove Sample Average | 0.549 |
|  | At Sample average or Above average | 0.567 |
| MASC-P | Belove Sample Average | 0.534 |
|  | At Sample average or Above average | 0.603 |
| PARS-severity mean score | Belove Sample Average | 0.706 |
|  | At Sample average or Above average | 0.391 |

*Note.* CAIS-P: Child Anxiety Impact Scale, Parent Report; CBCL-Anxious/Depressed subscale; MASC-P: Multidimensional Anxiety Scale for Children, Parent Version; PARS: Pediatric Anxiety Rating Scale severity mean score

| **DeLong tests on complete data** | | | |
| --- | --- | --- | --- |
| Measures | sub-group analysis | DeLong statistics | p values |
| CAIS-P | Below Sample Average versus Sample average or Above average | Z = -1.108 | p= 0.271 |
| CBCL-Anxious/Depressed | Below Sample Average versus Sample average or Above average | Z = -0.1227 | p = 0.903 |
| MASC-P | Below Sample Average versus Sample average or Above average | Z = -0.251 | p = 0.802 |
| PARS-severity mean score | Below Sample Average versus Sample average or Above average | Z = 1.029 | p = 0.305 |

Low: WISC-vocabulary subscale scores at or below the bottom 30%; Medium: WISC-vocabulary subscale scores between the middle 40%; High: WISC-vocabulary subscale scores at or above the top 30%

*Note.* CAIS-P: Child Anxiety Impact Scale, Parent Report; CBCL-Anxious/Depressed subscale; MASC-P: Multidimensional Anxiety Scale for Children, Parent Version; PARS: Pediatric Anxiety Rating Scale severity mean score

8.4.4. Comparisons of diagnostic accuracy across different sex

| Measures | Sex | Complete data diagnostic accuracy |
| --- | --- | --- |
| CAIS-P | Male | 0.719 |
|  | Female | 0.907 |
| CBCL-Anxious/Depressed | Male | 0.500 |
|  | Female | 0.546 |
| MASC-P | Male | 0.589 |
|  | Female | 0.673 |
| PARS-severity mean score | Male | 0.600 |
|  | Female | 0.759 |

*Note.* CAIS-P: Child Anxiety Impact Scale, Parent Report; CBCL-Anxious/Depressed subscale; MASC-P: Multidimensional Anxiety Scale for Children, Parent Version; PARS: Pediatric Anxiety Rating Scale severity mean score

| **DeLong tests on complete data** | | | |
| --- | --- | --- | --- |
| Measures | sub-group analysis | DeLong statistics | p values |
| CAIS-P | Male versus female | Z = -1.233 | p = 0.220 |
| CBCL-Anxious/Depressed | Male versus female | Z = -0.253 | p = 0.801 |
| MASC-P | Male versus female | Z = -0.308 | p = 0.759 |
| PARS-severity mean score | Male versus female | Z = -0.567 | p = 0.572 |

*Note.* CAIS-P: Child Anxiety Impact Scale, Parent Report; CBCL-Anxious/Depressed subscale; MASC-P: Multidimensional Anxiety Scale for Children, Parent Version; PARS: Pediatric Anxiety Rating Scale severity mean score

8.4.5. Comparisons of diagnostic accuracy for those younger than 10 years old, or 10 years old and above

We chose 10 years old as a cutoff for age because it was the closest year to both the upper and lower bound of 30 percentile and also the median.

| Measures | Age | Complete Data  Diagnostic Accuracy |
| --- | --- | --- |
| CAIS-P | <10 | 0.856 |
|  | >=10 | 0.719 |
| CBCL-Anxious/Depressed | <10 | 0.601 |
|  | >=10 | 0.557 |
| MASC-P | <10 | 0.688 |
|  | >=10 | 0.571 |
| PARS-severity mean score | <10 | 0.613 |
|  | >=10 | 0.642 |

*Note.* CAIS-P: Child Anxiety Impact Scale, Parent Report; CBCL-Anxious/Depressed subscale; MASC-P: Multidimensional Anxiety Scale for Children, Parent Version; PARS: Pediatric Anxiety Rating Scale severity mean score

| **DeLong test on complete data** | | | |
| --- | --- | --- | --- |
| Measures | sub-group analysis | DeLong statistics | p values |
| CAIS-P | <10 years versus >=10 years | Z = 0.857 | p = 0.393 |
| CBCL-Anxious/Depressed | <10 years versus >=10 years | Z = 0.273 | p = 0.785 |
| MASC-P | <10 years versus >=10 years | Z = 0.453 | p = 0.652 |
| PARS-severity mean score | <10 years versus >=10 years | Z = -0.076 | p = 0.939 |

*Note.* CAIS-P: Child Anxiety Impact Scale, Parent Report; CBCL-Anxious/Depressed subscale; MASC-P: Multidimensional Anxiety Scale for Children, Parent Version; PARS: Pediatric Anxiety Rating Scale severity mean score
